# An integrative atlas of chicken long non-coding genes and their annotations across 25 tissues

**DOI:** 10.1038/s41598-020-77586-x

**Published:** 2020-11-24

**Authors:** Frédéric Jehl, Kévin Muret, Maria Bernard, Morgane Boutin, Laetitia Lagoutte, Colette Désert, Patrice Dehais, Diane Esquerré, Hervé Acloque, Elisabetta Giuffra, Sarah Djebali, Sylvain Foissac, Thomas Derrien, Frédérique Pitel, Tatiana Zerjal, Christophe Klopp, Sandrine Lagarrigue

**Affiliations:** 1 PEGASE UMR 1348, INRA, AGROCAMPUS OUEST, 35590 Saint-Gilles, France; 2 SIGENAE Platform, INRA, 31326 Castanet-Tolosan, France; 3 Genotoul, INRA, US 1426 GeT PlaGe, Castanet-Tolosan, France; 4 GenPhySE UMR 1388, INRA, INPT, ENVT, Université de Toulouse, 31326 Castanet-Tolosan, France; 5 GABI UMR 1313, INRA, AgroParisTech, Université Paris-Saclay, 78350 Jouy-en-Josas, France; 6 IRSD, Université de Toulouse, INSERM, INRA, ENVT, UPS, Toulouse, France; 7 IGDR UMR 6290, Univ Rennes, CNRS, 35000 Rennes, France

**Keywords:** Genetics, Functional genomics, Gene expression, Gene regulation, Genome, Genomics

## Abstract

Long non-coding RNAs (LNC) regulate numerous biological processes. In contrast to human, the identification of LNC in farm species, like chicken, is still lacunar. We propose a catalogue of 52,075 chicken genes enriched in LNC (http://www.fragencode.org/), built from the Ensembl reference extended using novel LNC modelled here from 364 RNA-seq and LNC from four public databases. The Ensembl reference grew from 4,643 to 30,084 LNC, of which 59% and 41% with expression ≥ 0.5 and ≥ 1 TPM respectively. Characterization of these LNC relatively to the closest protein coding genes (PCG) revealed that 79% of LNC are in intergenic regions, as in other species. Expression analysis across 25 tissues revealed an enrichment of co-expressed LNC:PCG pairs, suggesting co-regulation and/or co-function. As expected LNC were more tissue-specific than PCG (25% vs. 10%). Similarly to human, 16% of chicken LNC hosted one or more miRNA. We highlighted a new chicken LNC, hosting miR155, conserved in human, highly expressed in immune tissues like miR155, and correlated with immunity-related PCG in both species. Among LNC:PCG pairs tissue-specific in the same tissue, we revealed an enrichment of divergent pairs with the PCG coding transcription factors, as for example LHX5, HXD3 and TBX4, in both human and chicken.

## Introduction

Since their description at the end of the 1990s^1,2^, long non-coding RNAs (LNC) have been shown to be involved in numerous biological processes, both at the cellular level (such as cell proliferation^3^ and differentiation^4^, metabolism^5^ and apoptosis^6^) and at the organism level, whether influencing sex differentiation^7^, agronomical traits^8,9^, disease^10^ or cancer^11^. LNC genes act through regulatory actions at every levels of gene expression, from the epigenetic modification of the chromatin^12^ to the regulation of transcription^13^ and protein translation^14^.

While it is well known that complex traits are mostly driven by variants located outside the coding regions^15^ that likely are regulatory variants modulating gene expression, the molecular chain of causality linking the DNA variant to the gene to the phenotype remains scarce. A better knowledge of LNC genes and of their regulatory functions could therefore be of help to identify their target genes, and possibly link them with the phenotype of interest. In model species such as human and mouse, LNC are well characterized with 15,137 and 10,177 LNC genes, respectively in Ensembl database (*versus* 20,465 and 22,604 protein coding-genes, respectively)^106^; although these estimates are nevertheless bound to increase^16^ in the continuity of works like the GENCODE v7 annotation of human LNC that manually annotated 9277 LNC in 2012^17^, while the human NONCODE (v5) integrated more than 96,000 LNC at the end of 2017^18^. In non-model species, such as farm species, LNC knowledge is largely incomplete. In the Ensembl release 94 reference database used in the present paper (October 2018), 4,643 LNC genes were known in chicken, none in cow and 361 in pig, *versus* 18,346; 19,994 and 22,452 PCG, respectively^16^. LNC annotation is one of the priorities of the Functional Annotation of ANimal Genomes (FAANG) initiative^19,20^. We and others recently reported LNC transcript sets for cattle, sheep, horse, pig, goat^20,21^ and chicken^22^.

Among farm species, chicken represents an interesting species, both for fundamental and applied research. It is used in evolutionary studies to evaluate the level of conservation of genomics feature among species^23^ and is a valuable model in the developmental biology research field^24^. Moreover, chicken is a species of great economic value, being one of the most consumed in the world, and representing a 300 billion dollars market^25^.

The first aim of this study was to enrich the chicken Ensembl gene catalogue in long non-coding genes. For this we used new LNC genes that were computationally predicted in this study using 364 RNA-seq samples, as well as LNC genes available in the public databases NCBI^26^, NONCODE^27^ ALDB^28^ and FR-AgENCODE^21,29^. This enriched gene catalogue can be useful for the scientific community working on chicken, especially for those aiming at analysing gene expression rather than modelling genes, and therefore use a reference annotation such as Ensembl or NCBI at the gene level.

The second aim of our study was the characterization of the identified LNC. We analysed their expression profiles in 25 distinct tissue types. Since LNC genes are generally very weakly expressed compared to the PCG^17,30^, we provide the level of expression for each LNC in the tissue in which it is the most expressed, giving an indication of how easy it is to study experimentally. Since the configuration of a LNC and its close PCG (hereafter, LNC:PCG pairs), such as the close divergent and antisense, can be an indicator of a regulatory role of the LNC on the PCG^31–34^, and therefore of an involvement in a common biological function^35^, we classified the LNC based on their genomic configuration with respect to their closest protein-coding gene (PCG) and screened for LNC:PCG pairs showing a significant co-expression. This latter approach is based on the “guilt-by-association” principle. It consists in grouping together genes (of known and/or unknown function) with a high expression correlation, with the hypothesis that this high correlation could be due to a common regulation, meaning that the genes play a role in the same biological process. We further searched for LNC hosting miRNAs genes, since the transcription of these LNC may result in the co-expression of their host miRNA^36^ and highlighted interesting cases suggesting that the LNC and the miRNA could participate to the same biological process. Finally, starting from the premise that a gene expressed in one or a few tissues likely plays a role related to these tissue functions^37^, we performed an in-depth analysis of LNC and PCG tissue specific expression. We showed that LNC are clearly more tissue-specific than PCG and pinpointed some specific features for the tissue-specific and divergent LNC:PCG pairs. The extended catalogue, in coordinates corresponding to the *Gallus_gallus-5.0* and the more recent *GRCg6a* assemblies, as well as all the information regarding the configurations of gene pairs and gene tissue-specificity are available in the Supplementary material of this article, and also on the FR-AgENCODE website (http://www.fragencode.org/). The files in this website will be regularly updated when new information or new version of the chicken genome assembly is available.

## Results

### Extension of ensembl gene catalogue with LNC gene models

**Figure 1:**
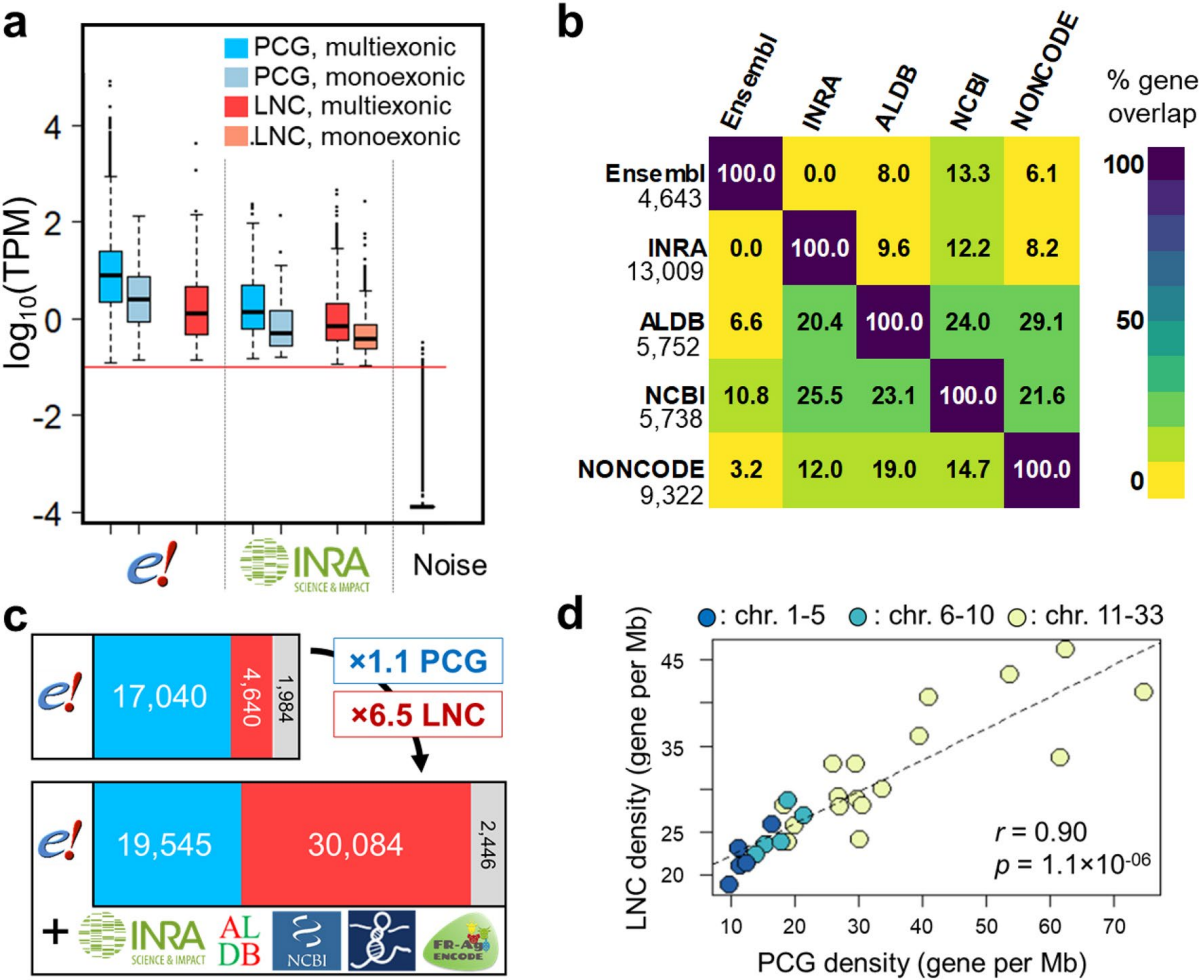
Extended gene catalogue features. (**a**) Expressions of the newly modelled genes compared to expression of the Ensembl genes and background noise, here given in the liver. The red line corresponds to the 0.1 TPM threshold. (**b**) Heatmap of the overlap between databases expressed in % of LNC (in line) shared among databases (in column), using 1 bp-or-more overlap. The number of LNC per database is mentioned in line. (**c**) The extended catalogue gathers 6.5 × more lncRNA genes and 1.1 × more PCG compared to Ensembl alone. (**d**) Correlation of LNC gene density to protein-coding gene density across the chicken macro-, medium- and micro-chromosomes. TPM: Transcript Per Million, chr: chromosome, Mb: Megabase.

The Ensembl gene catalogue (v94, December 2018) contained 24,881 genes (38,118 transcripts). Among these, 18,346 were annotated as protein-coding genes (PCG) and 4,643 as lncRNA (LNC) genes. Here we enriched this catalog by combining four complementary data sources: NCBI, ENCODE and ALDB LNC databases and the INRA catalogue newly generated in this study using 364 RNA-seq samples from 3 tissues. Using the pipeline “STAR – Cufflinks – Cuffmerge” and the “FEELnc” LNC prediction tool as described in Material & Methods section, we modelled 14,760 new genes composed of 1,199 PCG and 13,009 LNC genes. Among the LNC gene models, 7,265 were mono-exonics and 5,744 were multi-exonic. The expression of these new models was well above the background noise (Fig. 1a, “INRA” versus “Noise”) for both LNC and PCG, and for both mono-exonic and multi-exonic models, corroborating their existence. As expected, LNC models were less expressed than PCG models. We then combined this set of new models (the *INRAGALG* models) with the ones from NONCODE^27^, NCBI^26^ and ALDB^28^ public databases, to further extend the reference chicken Ensembl catalogue. The combination of these different catalogues is relevant since they are complementary as indicated by the low percentage of 1 bp-or-more overlapping transcripts between catalogues (from 3.2% to 29.1%) (Fig. 1b and Supplementary Figure S1 with more stringent overlapping criteria). The strategy used to add these four catalogues to the Ensembl catalogue is described in the Material and Methods. Briefly, we sequentially added the gene models from each database to a growing catalogue, keeping only the genes that had no same-strand 1 bp overlapping transcripts with a gene already present in the growing catalogue. The order of addition of the databases was determined using the CAGE peaks detected in 2017 by Lizio et al*.*^38^ that corresponds to the transcription start site (TSS) of the transcripts: we calculated the percentage of LNC transcript models having their 5′ extremity within ± 30 bp of a CAGE peak, suggesting a good modelling, at least in the 5′-end. The corresponding percentages were 7.3%, 5.5%, 5.3% and 4.2% for INRA, ALDB, NCBI and NONCODE respectively, giving the order of addition of these sources. For the ± 100 bp criteria, these percentages were slightly higher (11%, 10%, 10% and 7% respectively). These values are low when compared to the 41% and 50% for the PCG transcripts using the ± 30 bp and ± 100 bp criteria respectively. Overall, these results are consistent with those of Derrien et al*.*^17^ who found 15% for human LNC transcripts *versus* 55% for human PCG transcripts, using a ± 100 bp criteria. Lastly, we added to the catalogue, genes that we have recently produced in the multi-species FR-AgENCODE project^21,29^. The process leads to the generation of an extended catalogue of 52,075 chicken genes, with 30,084 LNC and 19,545 PCG genes, including all the Ensembl genes (4,643 LNC, 18,346 PCG and 2,446 other Ensembl genes, including the 1,705 small non-coding genes) (Fig. 1c). It gathers 6.5 times more LNC genes and 1.1 time more PCG, compared to Ensembl alone. This extended catalogue can be found in the form of a GTF file in Supplementary Data S1, and is available on the FR-AgENCODE project website^29^. The LNC density per chromosome was correlated with the density of PCG (Fig. 1d, Spearman *ρ* = 0.90, *p* = 1.1 × 10^−06^), with a higher density on the micro-chromosomes compared to the macro-chromosomes: 32 LNC and 36 PCG per Mb for the micro-chromosomes (chr 11 to 33) versus 22 and 12 per Mb respectively for the macro-chromosomes (chr 1 to 5).

### Gene expression across chicken tissues and classification of the LNC with respect to the closest PCG

**Figure 2:**
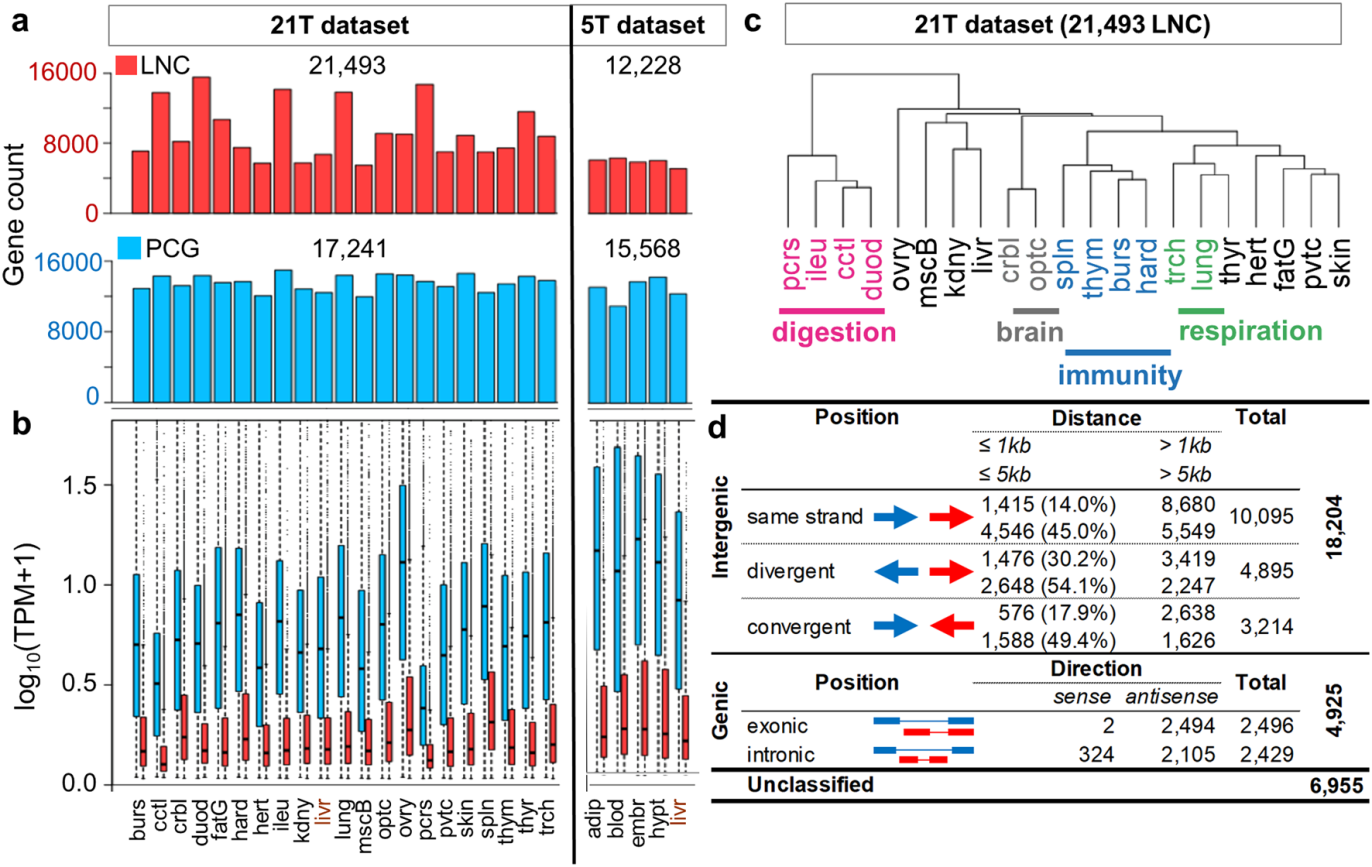
Overview of the extended catalogue in terms of expression and genomic configuration. (**a**) Number of LNC (red) and PCG (blue) expressed in each tissue: in the 21 T dataset, were expressed between 11,963 (muscle) and 14,983 (ileum) PCG with a median of 13,691 and between 5,492 (kidney) and 15,534 (duodenum) LNC with a median of 8794. In the 5 T dataset, were expressed between 11,024 (blood) and 14,313 (hypothalamus) PCG with a median of 13,165 and between 5127 (liver) and 6,319 (blood) LNC with a median of 6,043. (**b**) Boxplot of expression levels of LNC (red) and PCG (blue) in each tissue. (**c**) Hierarchical clustering of the 21 tissues from the 21 T dataset based on 21,493 LNC expressed in at least one tissue. Clustering performed using “1—Pearson correlation” distance and “ward” aggregation criteria. (**d**) Overview of the classification of LNC genes with respect to their closest PCG. For each configuration, the first line shows the numbers for a 1 kb threshold and the second line for a 5 kb threshold. Abbreviations in panels (**a**–**c**) stand for: burs: bursa of Fabricius, cctl: cecal tonsils, crbl: cerebellum, duod: duodenum, fatG adipose tissue around the gizzard, hard: harderial gland, hert: heart, ileu: ileum, kdny: kidney, livr: liver, lung: lung, mscB breast muscle, optc: optical lobe, ovry: ovary, pcrs: pancreas, pvtc: proventriculus, skin: skin, spln: spleen, thym: thymus, thyr: thyroid gland, trch: trachea.

Using the extended catalogue, we quantified the expression of the LNC and PCG models in the 21 and 5 tissue datasets, (hereafter called “21 T” and “5 T”, respectively). Out of the 52,075 genes of the extended annotation, on average 80% of the genes were expressed in at least one tissue of one dataset: 17,438 PCG (89%); 22,000 LNC (77%) and 777 other biotypes (32%). Interestingly, in the 21 T dataset similar numbers of expressed PCG were found across tissues (from 11,963 in muscle to 14,983 in ileum) while the number of expressed LNC are more variable (5,492 in kidney to 15,534 in duodenum) (Fig. 2a).

On average, LNC were 18 times less expressed than the PCG (Wilcoxon test, *p*-value < 2.2 × 10^−16^) (Fig. 2b), consistent with studies in human^17^, dog^39^ and chicken^22^. Among the 21,493 LNC and 17,241 PCG expressed in at least one tissue of the 21 T dataset, we observed 59% LNC (12,655) and 94%PCG (16,164) with expressions ≥ 0.5 TPM and 41% LNC (8,779) and 90% PCG (15,506) with expressions ≥ 1 TPM. The hierarchical clustering using the LNC expressions from the 21 T dataset shows biologically meaningful relationships among tissue types (Fig. 2c). The analysis grouped together tissues related to the nervous system (cerebellum and optical lobe), the immune system (spleen, thymus, bursa of Fabricius and the Harderian gland), the respiratory tract (trachea and lung, in green) and the digestive tract (pancreas, ileum, cecal tonsil and duodenum, in purple).

LNC models were classified with respect to the closest coding gene in the genome, according to the international gold-standard lncRNA classification provided by the GENCODE consortium^40^ and using the FEELnc classification tool^41^. Results are summarized in Fig. 2d; the details gene-by-gene can be found in Supplementary Data S2. Of the 30,084 lncRNA genes of our extended catalogue, 23,129 genes were classified, the rest being genes either located alone on a contig, or without PCG in a 100 kb window (named “unclassified”, see “Methods”). As expected, most LNC were intergenic (79%) and 21% were genic. Among the intergenics, the median distance between genes in the divergent LNC:PCG pairs was significantly lower compared to the distance within the convergent or same-strand pairs (3,957 bp *vs.* 5,130 bp, *p* < 4.73 × 10^−15^ and 3,957 bp *vs.* 6,005 bp, *p* < 2.2 × 10^−16^, Wilcoxon test). Splitting the intergenic genes in two classes based on distance^22^ (“close”: ≤ 5 kb and “distant”: > 5 kb) shows an enrichment of close divergent compared to the convergent or same strand genes (54.1% versus 49.4% and 45%; *p* < 3.9 × 10^−5^ and *p* < 2.2 × 10^−16^ respectively, Fisher test). These two observations regarding the divergent pairs are supportive of widespread bidirectional transcription.

### Co-expression differences of the LNC:PCG pairs according to their genomic configuration

**Figure 3:**
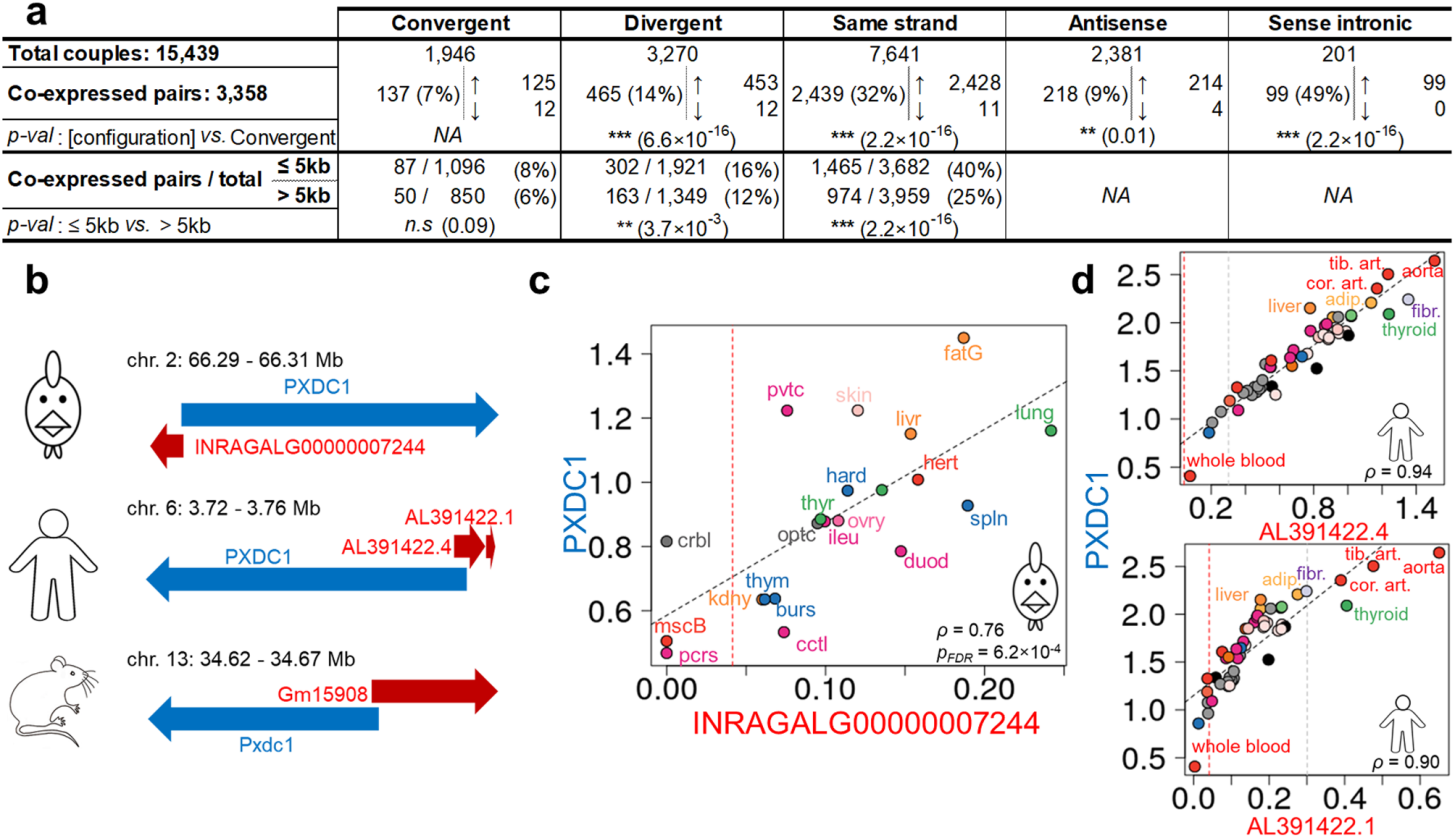
Classification and co-expression using the extended catalogue. (**a**) Overview of the correlations between the LNC and the PCG genes from all the expressed pairs, according to the different classes and the distance between the two genes of the pair. “*p-val: [configuration] vs. Convergent*” is the p-value of a Fisher test for the enrichment in significantly correlated pairs from the configuration in column *versus* the convergent configuration. “*p-val: ≤ 5 kb vs.* > *5 kb*”is the p-value of a Fisher test for the enrichment of significantly correlated pairs at less than *5* kb *versus* more than *5* kb. (*: *p-val* ≤ 0.05; **: *p-val* ≤ 0.01; ***: *p-val* ≤ 0.001; *n.s*: not significant). (**b**) Conservation of the genomic configuration of *PXDC1* and its closest LNC in chicken (top), human (middle) and mouse (bottom). In human, two close LNC are present. (**c**) Expression correlation in log_10_(TPM + 1) between INRAGALG00000007244 and PXDC1 across 21 tissues of the 21 T dataset. Tissues abbreviations are the same as in Fig. 2. (**d**) Conservation of the correlation between PXDC1 and its closest LNC in human in log_10_(TPM + 1). *NA* not applicable.

In order to detect biologically meaningful relationships between LNC and PCG, we studied the expression correlations across tissues of the LNC:PCG pairs using the 21 T dataset which had the higher number of tissues. The results are displayed on Fig. 3a. We found 15,439 pairs expressed in at least one tissue among the 23,129 classified pairs. Out of these, 3,358 had a significant correlation in terms of expression (absolute value of Spearman *ρ* ≥ 0.55, *p*_*FDR*_ < 0.05) with only 39 pairs having a negative correlation (Supplementary Table S1). We observed among the 3,358 co-expressed pairs a highly significant enrichment of divergent (14%), same-strand (32%) and sense intronics (49%) configurations, and a significant enrichment of antisense (9%) compared to convergent (7%). Furthermore, focusing on intergenic pairs, we observed a significant enrichment in co-expressed pairs separated by 5 kb or less compared to those separated by more than 5 kb for the divergent (16% versus 12%) and same-strand configurations (40% versus 25%) but not for the convergent configuration (8% versus 6%). Interestingly, comparing with co-expressed PCG:PCG pairs, we found 4,305 significantly co-expressed pairs, of which only 9 had a negative correlation. In these 4,305 pairs, we observed a significant enrichment in pairs separated by 5 kb or less versus more than 5 kb for all configurations, except convergent (Supplementary Table S2).

As an illustration, one example of positive correlation of LNC:PCG pairs is displayed in Fig. 3. The *PXDC1* (PX Domain Containing 1) gene shows a high expression correlation with the new INRAGALG00000007244 LNC model (Fig. 3c), which is in divergent configuration at 121 bp (Fig. 3b top). This pair is conserved in human and mouse (Fig. 3b, middle and bottom). In human, two close LNC genes are annotated in antisense and divergent configuration of *PXDC1*. Using the GTEx dataset^42^, we found that both human LNC genes display a high expression correlation with the human *PXDC1* coding gene (Fig. 3d, top and bottom). Their expression pattern and their proximity suggest that they may represent one single gene and not two as annotated. The LNC:*PXDC1* pair represents a nice example of a conservation across species of both the genomic configuration and co-expression. The existence of the new chicken INRAGALG00000007244 LNC model was confirmed by RT-PCR through a clear amplification using lung RNA (Supplementary Figure S2), followed by sequencing.

### LNC host small non-coding genes

**Figure 4:**
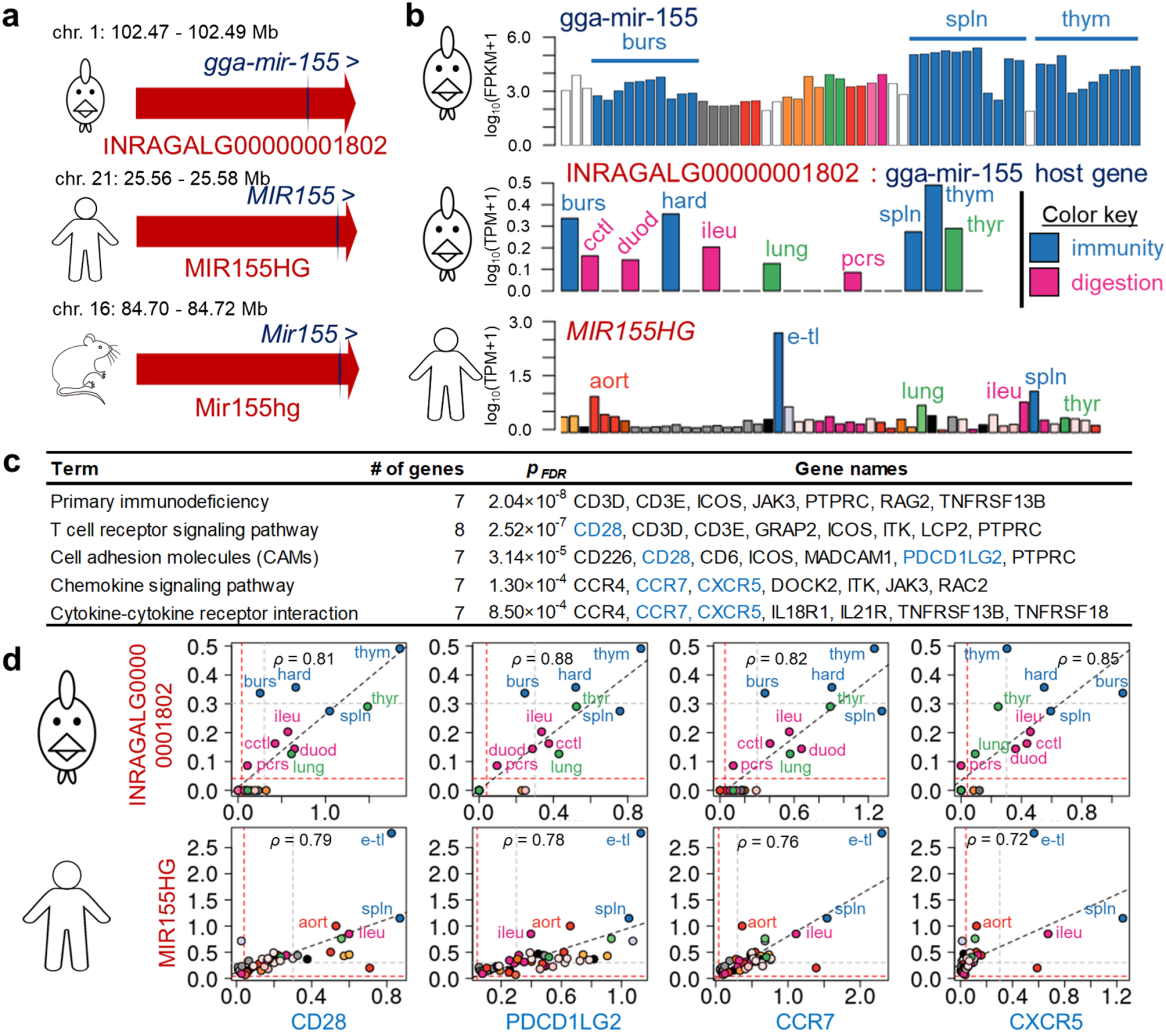
Conservation of genomic location and function of a miR host LNC across species. (**a**) INRAGALG00000001802 hosts gga-mir-155 and is conserved in human (MIR155HG) and mouse (Mir155hg). (**b**) Gga-mir-155 (top) and its host LNC, INRAGALG00000001802 (middle), are mostly expressed in immunity-related tissues in chicken, similarly to MIR155HG in human (bottom). Gga-mir-155 expression is expressed in log_10_(FPKM + 1) and the 55 Chickspress database tissues are ordered as the list available in Supplementary Table S22A, INRAGALG00000001802 and MIR155HG expressions are expressed in log_10_(TPM + 1) and the 53 GTEx project tissues are ordered as the list available in Supplementary Table S22B (**c**) Top 5 enriched KEGG terms supported by more than 5 genes associated to the PCG correlated to INRAGALG00000001802. PCG in blue are used in next panel. (**d**) Co-expression of four PCG from previous panel with INRAGALG00000001802 in chicken (top) or MIR155HG in human (bottom). Abbreviations in panels (**b**) and (**d**) stand for: aort: aorta, burs: bursa of Fabricius, cctl: cecal tonsils, duod: duodenum, e-tl: EBV-transformed lymphocytes, hard: harderial gland, ileu: ileum, lung: lung, pcrs: pancreas, spln: spleen, thym: thymus, thyr: thyroid gland.

We classified miRNAs and other small RNAs (Small nucleolar: snoRNAs, and small nuclear: snRNAs) genes with their closest LNC using FEELnc, in order to find LNC that host such genes. We found that 0.6% of the LNC (185 out of 30,084) hosted one or more miRNA, and that 16% of miRNA (177 out of 1,116) were hosted by one or more LNC. This is consistent with the literature in human in which 17.5% of the miRNA are located in LNC^36^. Similarly, for small RNAs, we identified 42 LNC (0.14% of total) hosting 58 small RNA genes, of which 48 snoRNAs (19% of snoRNAs) and 10 snRNAs (9% of snRNAs). These results are provided in Supplementary Data S2. Focusing on the 185 LNC hosting miRNA, we studied their co-expression with PCG across the 21 tissues of the 21 T dataset. Out of these 185 host LNC, 126 were expressed in at least one tissue of the 21 T dataset, and had between 0 and 939 correlated PCG among the whole dataset (spearman | *ρ* |≥ 0.8, *p*_*FDR*_ ≤ 0.01), all positively. The selection process described in Material and Method left a list of 10 miRNAs cited in the literature and for which and 75% of the correlated PCG had a HGNC (Supplementary Tables S3–S13). Among these 10 host LNC, we found two cases in which the host LNC was conserved in human and mouse, and the correlated PCG were enriched in functions similar to those of the miRNA. The first example is reported in Fig. 4. The LNC INRAGALG00000001802 hosts the gga-mir-155, in a similar genomic configuration as MIR155HG and Mir155hg that host MIR155 and Mir155 in human and mouse, respectively (Fig. 4a). In chicken, we found that gga-miR-155 was highly expressed in the spleen and to a lesser extent in the thymus using the expression data from Chickspress^43^ (Fig. 4b, top). In the 21 T dataset, INRAGALG00000001802 is mostly expressed in immunity-associated tissues (*i.e.* bursa of Fabricius, Harderian gland, spleen and thymus) and to a lesser extent in digestive system-associated tissues (caecal tonsil, duodenum, ileum and pancreas), as showed in Fig. 4b, middle. A similar pattern was observed in human for MIR155HG using the GTEx data, with a notable expression in the lymphocytes and the spleen (Fig. 4b, bottom). The 118 PCG highly correlated with INRAGALG00000001802 (ρ ≥ 0.8) had as top5 enriched KEGG terms, terms associated with immunity (Fig. 4c). Figure 4d, top, provides some chicken LNC:PCG co-expression for four immunity-related genes taken as example. Interestingly, their human 1-to-1 orthologues were also well-correlated with the MIR155HG LNC, with the highest expression in the immunity-related tissues (Fig. 4d, bottom), suggesting that the mode of action responsible for this correlation in chicken is conserved in human. Interestingly, we found among the 118 PCG only two targets of the human hsa-miR-155-5p (*LAT2* and *ITK*), identified in both miRTarBase (with support type indicated as “weak”) and mirDB, and one target of human hsa-miR-155-3p (*TXK*) using mirDB. *ITK* was also identified as a target of the chicken gga-miR-155 in mirDB (target prediction score = 98). The new chicken INRAGALG00000001802 LNC model was confirmed by RT-PCR through a clear amplification using spleen RNA samples (Supplementary Figure S2), followed by sequencing. The second case concerned gga-mir-124a-2, hosted in sense of intron by NONGGAG008930, which was expressed in the optical lope and cerebellum, and was correlated with 89 PCG enriched in genes associated to the development of the nervous system. In human, MIR124-3, of which gga-mir-124a-2 is a one-to-many ortholog with the same synteny, is hosted in antisense of intron by a LNC expressed in brain parts and testis in the GTEx dataset. In mouse, *Gm27032* and Mir124a-3 are located on the same strand and have a small overlap. This LNC was well correlated with the human 1-to-1 ortholog of some of the 89 chicken PCG correlated with NONGGAG008930. Using Chickspress data, we found that gga-miR-124a, in particular the − 3p transcript, was expressed in cerebellum, cerebrum and hypothalamus of adult chicken. Among these 89 PCG, we found only 3 targets of the chicken or human miRNAs. All these information are summarized in Supplementary Figure S3.

### LNC are more tissue-specific than PCG

**Figure 5:**
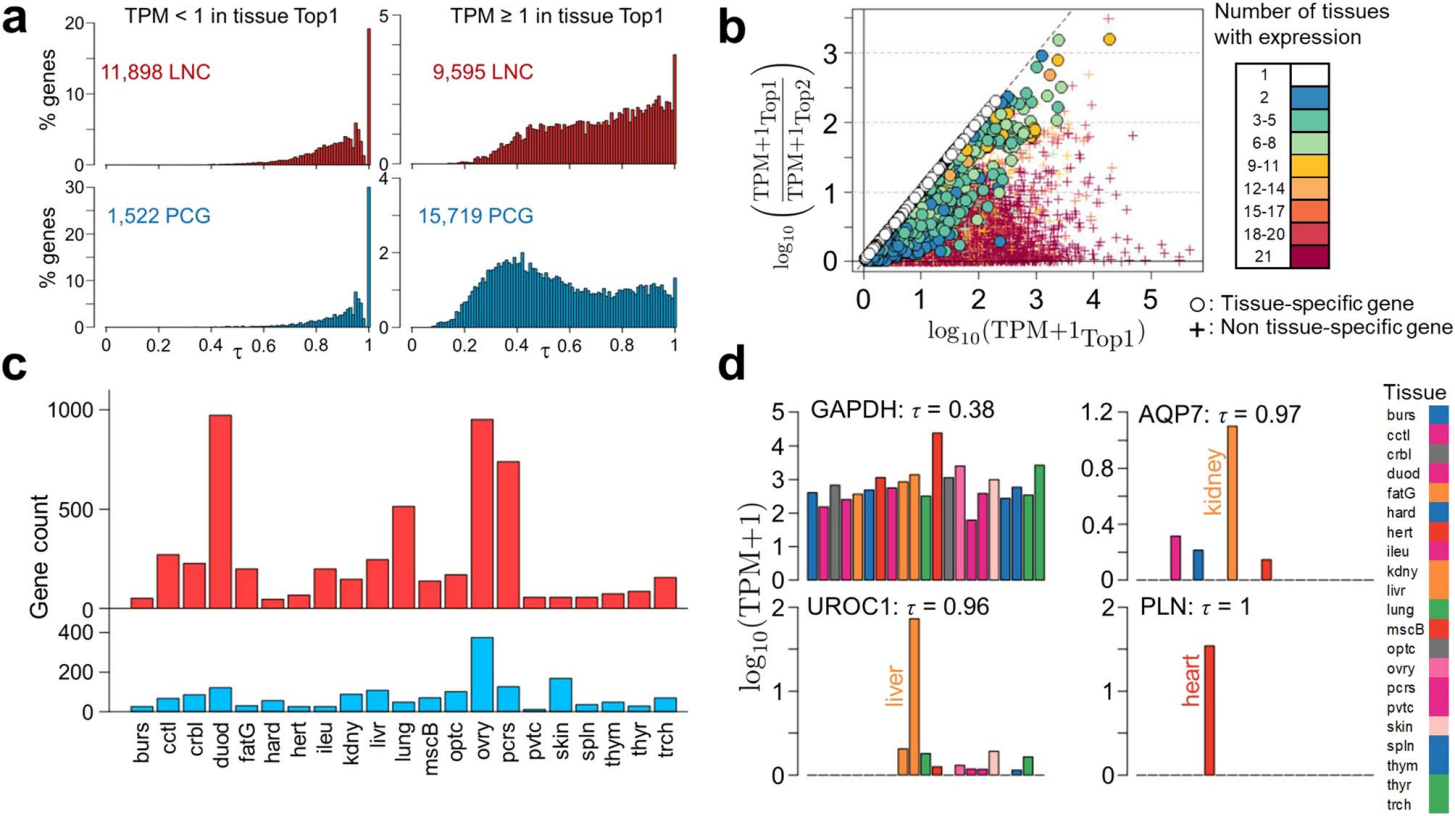
Overview of the tissue-specificity of PCG (in blue) and LNC (in red) in the 21 T dataset. (**a**) Distribution of τ values for LNC (top row) and PCG (bottom row) for two expression levels: TPM < 1 (left column) and TPM ≥ 1 (right column). The total number of corresponding genes is indicated in each plot. (**b**) For all the expressed genes (LNC and PCG), ratio of expression between the tissue with the highest expression (“Top1”) and the second tissue with highest expression (“Top2”) as a function of the expression in the tissue Top1. Colour indicates the number of tissues in which each gene is expressed, and the shapes differentiate the genes with a τ ≥ 0.95 (dots) versus τ < 0.95 (crosses). (**c**) Number of LNC (red) and PCG (blue) which are tissue-specific (τ ≥ 0.95) in each of the 21 tissues. (**d**) Expression profiles of 4 genes: the ubiquist *GAPDH* (top left), the tissues-specific *AQP7* and *UROC1* (top right and bottom left) and the heart-exclusive *PLN* (bottom right), expressed only in the heart. Tissues abbreviations are the same as in Fig. 2.

We studied tissue-specificity using the τ metrics^44^, shown by Kryuchkova et al*.*^45^, to have a good correlation between datasets, a good biological relevance and to be robust to normalization methods, compared to other metrics such as the Gini coefficient^46^ or PEM^47^. The τ metrics associates to each gene a score ranging from 0 to 1. Zero corresponds to a gene that would be expressed at the same level in every tissue, while 1 corresponds to a gene that is expressed only in one tissue. Genes with a τ close to 1 usually show an important expression difference between the tissue with the highest expression and the other tissues in which it can be also expressed. It is noteworthy that the relevance of an analysis related to tissue specificity depends on the number of tissues studied. That is why we conducted this study using the 21 T dataset composed of a high number of tissues. Figure 5a shows the repartition of the τ values of the LNC (top row) and PCG (bottom row) for two expression levels: TPM < 1 in the tissue with highest expression (left) or TPM ≥ 1 in the tissue with highest expression (right). For the LNC, 55% of the genes had TPM < 1 and 45% had TPM ≥ 1, while these proportion were 9% and 91% for the PCG. For both the LNC and the PCG with TPM < 1, the distribution of the τ values is clearly right-leaning, showing only one hump close to 1 and a high number of genes with a τ equal to 1. With TPM ≥ 1, PCG and LNC show different τ-values distributions. For the PCG (Fig. 5a, bottom right), we clearly observe two peaks around τ = 0.4 and τ = 0.95, and a peak at τ = 1. The first hump corresponds to relatively ubiquitously expressed genes, with among them the well-known housekeeping gene Glyceraldehyde-3-Phosphate Dehydrogenase (*GAPDH*, τ = 0.38). The second hump corresponds to tissue-specific genes, with for example the Aquaporin 7 (*AQP7*, τ = 0.97) or the Urocanate Hydratase 1 (*UROC1*, τ = 0.96). Finally, the peak at τ = 1 corresponds to genes expressed in only one tissue (tissue-exclusive), such as the Phospholamban (*PLN,* τ = 1) expressed only in the heart, which is the principal regulator of the Ca2/-ATPase of cardiac sarcoplasmic reticulum^48^. The LNC (Fig. 5a, top right) presented a distribution of the τ values that is clearly right-leaning, showing only one hump close to 1 and a high number of LNC with a τ equal to 1. Interestingly, the same patterns were observed for both LNC and PCG with expression data across 26 tissues from the dog species^39^ (without consideration for the expression level, see Supplementary Figure S4). These distributions suggest a higher tissue-specificity for the LNC than the PCG: 6% of PCG with TPM ≥ 1 and 13.4% of LNC with TPM ≥ 1 had a τ value ≥ 0.95. We further characterized the genes having a tissue-specific expression, i.e. with a τ ≥ 0.95, a stringent threshold of tissue-specificity^39^ by analysing at the same time their expression across tissues and the number of tissues in which they are expressed. The Fig. 5b shows for all genes the expression difference between the tissue with the highest expression (named “Top1”) and the second tissue with highest expression (named “Top2”) on the Y-axis, as a function of the expression in the Top1 on the X-axis. The number of tissues in which each gene is expressed is given by the colour of the dot. Most of the tissue-specific genes are expressed either in only one (n = 3,427 genes, white dots) or in a few tissues (2 to 5 tissues, n = 3,579 genes). The few genes that are expressed in 9 to 11 tissues (n = 62, yellow dots) or even 12 to 16 tissues (n = 13, orange/red dots) show a high level of expression in Top1 and are close to the diagonal, where Y = X, meaning that the difference between Top1 and Top2 is close to the value of Top1, *i.e.* their expression in Top2, and therefore in all the other tissues, is very weak (Supplementary Table S14). Overall, we found that 25.2% of the LNC (n = 5,422) and 9.8% of the PCG (n = 1,713) were tissue-specific in the 21 T dataset. Interestingly, the analysis of the 5 tissues in the 5 T dataset, revealed similar patterns for LNC and PCG, with 43.1% (n = 5,271) and 8.4% (n = 1,150) of tissue-specific genes, respectively (see Supplementary Figure S4). As expected, these percentages of tissue-specific genes are higher than what was observed in the 21 T dataset, due to the smaller number of tissues in the 5 T dataset. In each tissue, we found between 11 (proventriculus) and 375 (ovary) tissue-specific PCG with a median of 67 (Supplementary Table S14), and between 46 (harderian gland) and 972 (duodenum) tissue-specific LNC with a median of 156 (Fig. 5c, Supplementary Table S15). For each tissue, we realized a gene ontology (GO) terms enrichment analysis with the tissue-specific PCG (Supplementary Table S16). For example, the ovary-specific genes (tissue with the most tissue-specific PCG, see Fig. 5c) were enriched in 155 biological processes GO terms, of which “reproductive process” (GO:0,022,414, *p*_*FDR*_ ≤ 9.60 × 10^−15^) or “sexual reproduction” (GO:0,019,953, *p*_*FDR*_ ≤ 5.75 × 10^−8^). In the heart, the heart-specific genes (second tissue with the most specific PCG, see Fig. 5c) were enriched in 48 GO terms, of which “cardiac muscle tissue development” (GO:0,048,738, *p*_*FDR*_ ≤ 2.55 × 10^−9^) or “blood circulation” (GO:0,008,015, *p*_*FDR*_ ≤ 1.7 × 10^−3^). The expressions of three tissue-specific and one ubiquitously expressed are shown in Fig. 5d. *GAPDH* (most left) clearly shows a tissue-ubiquist expression pattern, with overall similar levels of expression across all tissues, albeit with a peak in muscle consistently with the literature^49^. *AQP* and *UROC1* have similar τ values, even though the former is expressed in four tissues while the latter is expressed in 10 tissues. The high difference in expression level between the Top1 and Top2 tissues of *UROC1* (liver and kidney, respectively) explains its tissue-specific classification. Finally, *PLN* clearly shows a tissue-specific pattern, being expressed in the heart only. More generally, for the tissue-specific genes (τ ≥ 0.95), the mean expression fold change between the Top1 and Top2 tissues is equal to 4.

We then investigated the liver-specific genes, this tissue being present in both 21 T and 5 T datasets. We found 247 (94 PCG; 145 LNC and 8 other biotypes) and 1,307 (326 PCG; 956 LNC and 25 other biotypes) liver-specific genes in the 21 T and 5 T datasets, respectively. We found that 208 genes (71 PCG and 130 LNC) were common to both datasets, meaning that the liver-specific genes from the 21 T dataset are almost included in those of the 5 T dataset. These genes are available in the Supplementary Table S17. Keeping only the genes with a unique HUGO identifier, the KEGG term enrichment analysis of the 54 common PCG revealed six KEGG terms (*p*_*FDR*_ ≤ 0.05) provided in Supplementary Table S18, all of them related to liver function.

### Tissue specific pairs in antisense or divergent configurations are enriched in DNA-binding transcription factors

**Figure 6:**
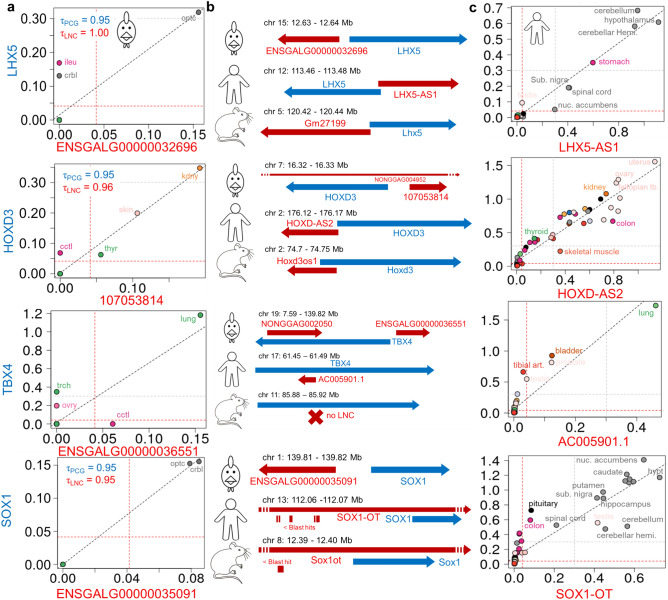
Tissue expression in chicken (**a**) and human (**c**) for 4 LNC:PCG pairs with similar genomic configurations between the two species (**b**). (**a**) Log_10_(TPM + 1) expression of the LNC (X-axis) and the PCG (Y-axis) (top) for chicken across the 21 tissues of the 21 T dataset for the four chicken divergent pairs for which both members are tissue-specific in the same tissue. (**b**) Genomic configuration in three species of the LNC:PCG pairs. In red the LNC, in blue the PCG. For the LNC:SOX1 pair, were added the positions of hits (in minus strand) resulting in the mapping of ENSGALG00000035091 chicken LNC sequences to human and mouse genome on the opposite strand. (**c**) Expression in log_10_(TPM + 1) of LNC:PCG pairs in 53 human tissues using the GTEx data. Tissues abbreviations are the same as in Fig. 2.

Combining the tissue-specificity of both LNC (n = 5,422) and PCG (n = 1,713) from 21 T dataset with the classification of LNC:PCG pairs, we found 100 pairs for which both members were tissue-specific and had the same “Top1” tissue (Supplementary Table S19). The GO term analysis of these 100 genes (of which 45 PCG with a known identifier) revealed molecular functions related to “DNA-binding transcription factor activity, RNA polymerase II-specific” (GO: 0,000,981), supported by 14 genes: *ALX4*, *BARHL1*, EMX1*, HMX1*, HOXD3*, LHX5*, *MNX1*, SOX1*, TBX4*, *TBX5*, *TLX1*, *ZIC1*, *ZIC2* and *ZIC4* (Supplementary Table S20)*.* Interestingly, these genes are enriched in the divergent configuration. In fact, in the 100 pairs list, only 12 genes belonged to the divergent class and six of them (indicated in the text with an asterisk) are related to the GO term abovementioned. Five out of the 6 LNC:PCG pairs seem to be conserved as antisense or divergent configurations in human and/or mouse as shown in Fig. 6b for *LHX5*, *HOXD3*, *TBX4* and in Supplementary Figure S5 for *EMX1* with the human ENSG00000278060 LNC, and *Hmx1* with the mouse ENSMUSG00000055944 LNC. Note that for *SOX1*, the closest LNC of human and mouse *SOX1* is an LNC-OT, i.e. an LNC in the same strand of the *SOX1* gene whereas in chicken both members of the LNC:*SOX1* pair are in opposite strands. Even if the LNC sequences are lowly conserved between distant species^50^, we found six significant blast hits of length 41 to 253 in human and one sequence of length 296 bp in mouse similar to the one of chicken *SOX1* divergent LNC at 5380 bp and 4184pb, respectively, upstream of human and mouse *SOX1* gene on the opposite strand (Fig. 6b, bottom, Supplementary Table S21). Using the GTEx data^42^, we analysed the co-expression *LHX5*, *HOXD3*,*TBX4* and SOX1 pairs available, the two other genes, *EMX1* and *Hmx1*, being absent in the GTEx dataset. Expression correlation of these four pairs in chicken and human, as well as their configurations are displayed in Fig. 6a and Fig. 6b respectively. The four chicken LNC models were confirmed by RT-PCR through a clear amplification using hypothalamus RNA samples for ENSGALG00000032696 and ENSGALG00000035091, kidney RNA sample for 107,053,814 and lung RNA sample for ENSGALG00000036551 (Supplementary Figure S2), and sequencing.

### Conversion of the extended catalogue content to the GRCg6a newest version of the chicken genome assembly and the associated Ensembl v100 annotation

Since this work proposes a GTF build on the Ensembl v94 annotation in *Gallus_gallus-5.0* genome coordinates, we also generated an extended version of the Ensembl v100 annotation of the new *Gallus gallus* genome reference (*GRCg6a*), comprising genes present in our present catalogue that did not overlap genes from the Ensembl v100 annotation. The methods used for this update are described in details in the Material and Methods section. As a result, we added to the 24,356 genes of the Ensembl v100 annotation (16,878 PCG; 5,506 LNC; 1,972 others) 18,994 LNC gene models from the databases presented in this work, marked in the Supplementary Data S2. In addition, 3,179 models from the v94 annotation that were removed but that were mapped in the *GRCg6a* genome assembly. As a result, we generated an annotation comprising a grand total of 46,529 genes. Hence, users wishing to work on *GRCg6a*, can use this second GTF file available on the FR-AgENCODE website (http://www.fragencode.org/) to benefit from the Ensembl v100 enriched in LNC annotation.

## Discussion

This work provides to the community an extended, LNC-enriched, gene catalogue of the chicken genome composed of 30,084 LNC, 19,545 PCG and 2,446 others genes, for a total of 52,075 genes. These data, in the form of a GTF file, are provided in Supplementary Data S1, and are ready to use by the community for RNA-seq expression analyses. We also provided a wealth of information in a table file (Supplementary Data S2 and its “read me” file, Supplementary Data S3). First, the configuration of the 30,084 LNC with respect to their closest PCG, plus the name of the PCG associated as well as the genomic distance between them. Second, for the 40,215 genes (of which 22,000 PCG and 17,438 LNC) expressed in one or both 21 T and 5 T datasets, we provide information relative to the tissue-specificity: the number of tissues in which the gene is expressed, the expression (in TPM) of the Top1 and Top2 tissues, the names of these tissues and the tissue-specificity values (τ values) in both datasets. For the LNC:PCG configurations, we also provide the spearman correlation of the expression for each gene pairs across the 21 and the 5 tissues of both datasets. All this information can be precious for researcher interested in one or several LNC to infer hypotheses about their expression and function. This GTF file, build on Ensembl v94 annotation, in Gallus_gallus-5.0 coordinates, and all related information are available on the FR-AgENCODE website (http://www.fragencode.org/), and they will regularly be updated, in particular when new assemblies are published. As a first update, we propose in this website an extended version of the Ensembl v100 annotation of the new *Gallus gallus* genome reference (GRCg6a), comprising genes present in this catalogue that did not overlap genes from the Ensembl v100 annotation, in GRCg6a coordinates.

We then performed different types of analysis in order to provide some functional annotation to these LNC. The analysis of the LNC:PCG configurations revealed different types of pairs, in proportions that were consistent with the literature in human^17^ and in farm species (pig, cattle and chicken)^51^, with a majority of intergenic genes (approximately 80%) of which 56% are in same-strand, the rest being in divergent and convergent configurations. Pairs in same-strand configuration should be considered with caution, since it is possible that the LNC is in fact a part of a not yet properly modelled PCG, especially when the gene couple is significantly co-expressed across tissues (as we show for 32% of the same strand pairs). Indeed, in non-model species, PCG isoforms are still poorly annotated. Only 38,118 isoforms are described in the chicken Ensembl v94 annotation for 24,881 genes in total, while more than 200,000 transcripts are modelled for 57,720 genes in total in the human Ensembl v94 catalogue. As an example, we recently showed that an LNC located at 978 bp in same-strand of *SCD1* was in fact part of this gene^52^. However, this situation is also true for close, same-strand LNC, as exemplified in Fig. 3d, in which the high correlation of the two human LNC with the PCG genes suggests that they in fact constitute one unique gene.

The divergent or genic configuration for LNC:PCG pairs associated to their co-expression may allow to formulate hypotheses on the function of LNC. Indeed, a significant expression correlation between two genomically close genes is an argument for the existence of a common regulation^53^ or even a regulation of the PCG by the LNC^35^. In both cases, we can hypothesize that the LNC is involved in the same biological processes as the PCG^54,55^. These hypotheses inferred by co-expression combined to gene configurations should allow to better orientate molecular biology experiments in order to better understand the biological role of the LNC of even of the LNC:PCG pair. Interestingly, we found a significant enrichment of co-expressed pairs among the divergent LNC:PCG configuration (14% of these pairs) and among the sense of introns configurations (49% of these pairs) compared to the convergent configuration (7% of these pairs). This enrichment is more limited but remains significant for the antisense configuration with 9% of these co-expressed pairs, compared to the convergent configuration. Divergent pairs suggests the presence of a bidirectional promoter that regulates both genes^56^, or even an effect of the LNC on the PCG expression^31,32^, even though the precise mechanisms remain unclear. We highlighted an example with the divergent LNC:*PXDC1* pair, conserved between chicken and mammals. *PXDC1* is a gene for which the function is still poorly known. It was found to be repressed in the liver of mice and rats exposed to a pollutant of the dioxin class (TCDD, or 2,3,7,8-tetrachlorodibenzo-p-dioxin)^57^. Furthermore, it was shown that transcriptional activation of *PXDC1* using a CRISPR activation system increased the survival of an acute myeloid leukaemia cell line exposed to cytarabine, a molecule used in standard chemotherapeutic mix for this leukaemia, hence increasing the resistance of the cells to the chemotherapy^58^. The high expression correlation between *PXDC1* and its divergent LNC suggests that the latter plays a role in the same biological processes as *PXDC1*. Antisense LNC, for their part, have also been shown to regulate their host gene expression, at both the transcriptional level, by the inducement of chromatin remodeling^33^, or at the post-transcriptional level^34^. Finally, sense intronic can arise from the splicing of a PCG or be produced from independent transcriptional unit^59^. They may regulate the expression of their host PCG, which are also associated to transcription regulation^60^, as shown by Guil et al*.*, who observed a down-expression of the gene *SMYD3* with the over-expression of a LNC from its intron^61^. In this study, we described 214 and 99 significantly co-expressed LNC:PCG pairs in antisense and sense intronic configuration respectively.

Interestingly, across all the LNC:PCG configurations, we found only a small number of pairs for which the correlation was significantly negative with 1.2% (39 pairs) out of the 3,355 significant correlations (with 0 for sense intronics and 12 for both the divergent and convergent configurations). Such a result was also observed in human^17^, for which 0.11% of the pairs LNC:PCG showed correlation lower than − 0.5, and in dog^39^, for which this percentage was 0.71%. Taken together, these results shared among different species suggest that LNC tends to act as positive, rather than negative, regulators or cofactors of the transcription of their closest gene, although there are well-known examples, such as *HOTAIR*^62^ or *XIST*^63^, that show that LNC can induce gene silencing. Interestingly, this trend was also found in PCG:PCG couples, for which only 0.15% (6 pairs) out of the 4,125 significant correlations were negative (3 divergent and 3 same-strand).

We found in chicken the same proportion of miRNA hosted by LNC than in human, 16% compared to 17.5%^36^. LNC hosting miRNA are thought to act as pri-miRNA (coined lnc-pri-miRNA in Dhir et al*.*^36^), which are the precursors of pre-miRNAs, themselves precursors of miRNAs^64^. Among the 10 LNC hosting miRNA chosen here to be deeply analysed because of their functional annotation in the literature, we found two cases in which the LNC hosting a miRNA might be associated to the same biological process as the miRNA. Indeed, we observed that mir-155, which is involved in the normal immune function^65^ and also expressed in immune-related tissues in chicken is hosted by a LNC that we also found to be highly expressed in the immune-related tissues and correlated in expression with multiple immunity-related genes in both chicken and human. Such results are consistent with a recent work conducted by Maarouf et al*.*^66^ who showed in a human cell line that its host LNC, MIR155HG participate to the immune response to the influenza A virus (IAV). What is more, Maarouf et al*.*^66^ showed that MIR155HG deleted of the sequence of MIR155 still significantly suppressed the IAV replication, clearly indicating that this LNC had an action on its own, not only by harbouring MIR155 sequence. While the immunity-related function of miR-155 and now of the LNC hosting this miR are known, the relationships between these two genes is not clear. The low number of miR-155 targets detected among the correlated genes tends to suggest that mir-155 does not directly act on the immunity-related PCG co-expressed with its LNC. The second example concerned gga-mir-124a-2, the human ortholog of which is MIR124-3, which is involved in neurogenesis^67,68^. We observed that gga-mir-124a-2 was expressed in different brain-parts in adult chicken, and found that its LNC hosting gene in chicken and human was also expressed in the brain-related tissues. In both species, the LNC were also correlated in expression with genes involved in the nervous system. Supplementary experiments are needed to better understand mechanisms underlying such PCGs and miR155 host LNC or of miR124 host LNC co-expression across tissues.

Our analysis of the tissue-specificity of the gene expression showed that LNC are more tissue-specific compared to PCG: 25.2% of LNC versus 9.8% of PCG for the dataset with 21 tissues, using the τ metrics with a threshold set at 0.95. The tissue-specificity of genes depends on multiple factors, such as the number of tissues analysed or the tissue-specificity metrics used. Nevertheless, the higher tissue-specificity of LNC compared to PCG is consistent with previous works conducted on several species, even if the ratio of tissue-specific genes in each biotype is variable. In human, Derrien et al*.*^17^, using 16 tissues and counting the number of tissues in which each gene was expressed, found that approximately 27% of the LNC and 4% of the PCG were expressed in one or two tissues, while Cabili et al*.*^30^, using 24 tissues and cell types, found more extreme values with 78% of LNC and 19% of PCG found to be tissue-specific using an entropy-based measure. Melé et al*.*^69^, using the GTEx data composed of 43 healthy tissues and 13 brain subregions of cells lines also commented on the fact that PCG are generally ubiquitous while LNC are more typically tissue-specific. In dog, Le Béguec et al*.*^39^, using 26 tissues and the τ metrics with a threshold set at 0.95, found that 44% of LNC and 17% of PCG were tissue-specific. It is noteworthy that the absence of the testis in our analysis may be responsible for an under-estimation of the total number of tissue-specific genes, since this tissue is well known for having a specific expression. Indeed, Melé et al*.*^69^ found that ~ 95% of the approximately 200 genes expressed in only one tissue were expressed in the testis.

Concerning the liver, which is the only tissue common to both independent 21 T and 5 T datasets analysed in this work, the high intersection of liver-specific genes detected between both datasets shows a good reproducibility of the results. This result shows the robustness of the gene tissue-specificity despite the technical and biological variations between the two datasets, from RNA extraction to sequencing, and from lines to ages or breeding conditions. These common genes were in enriched liver functions as “Complement and coagulation cascades”^70^, “Metabolic pathways”, “Histidine metabolism”^71^, “Tryptophan metabolism”^72^, “Steroid hormone biosynthesis”^73^ and “PPAR signalling pathway”^74^.

Interestingly, we observed an enrichment of GO terms related to the regulation of transcription among the LNC:PCG pairs for which both members were tissue-specific in the same tissue, with an over-representation of the divergent pairs. Five LNC:PCG pairs for which the PCG codes for a transcription factor involved in embryonic development or cell lineage seemed to be conserved between chicken and mammals. First, they share the same divergent or antisense configuration between chicken and human and/or mouse, at the exception of SOX1 for which a LNC was found in divergent configuration in chicken, versus in sense of intron overlapping in human and mouse (called *SOX1-OT*), but for which blast hits were detected in divergent configuration, suggesting the existence of another LNC. Hence, it is difficult to conclude on the conservation of ENSGALG00000035091 even if a high co-expression and similar tissue-specificity between the human *SOX1* and *SOX1-OT* genes was observed. Second, the tissue specificities observed in chicken are consistent with the one observed in human using GTEx data for *LXH5* and *SOX1* as brain-specific, *TBX4* as lung-specific and *EMX1* as kidney-specific. *HMX1* is found to be skin-specific in chicken while it is testis and brain specific in human. Interestingly, we found *HOXD3* to be kidney-specific in chicken while in human its “top tissues” are part of the female reproductive tract, just before the kidney (Fig. 6c,). This tends to indicate an expression conservation within the kidney across species, but not in the reproductive tract between these two species diverging on reproductive functions (oviparity versus viviparity). Third, these common tissue specificities are consistent with the function of the proteins associated to the PCG of each couple. *LXH5* and *SOX1* have been shown to be involved in neural determination or differentiation^75,76^. In particular, *SOX1* is known to control the development of the neural ectoderm from which the optical lobe and cerebellum are derived in adults. *HOXD3* belongs the HOXD cluster known to regulate the metanephric kidney development in mammals^77,78^. Recently, *EMX1* was reported as a novel nephron segment regulator during embryonic kidney development^79^. Finally, different studies report an important role of *TBX4* in lung development^80–82^.These results raises the question of the precise role of the LNC on the PCG in each of these tissue-specific pairs in their respective tissues.

Overall, the ability to obtain a comprehensive catalogue of the LNC in a species depends mainly on the number of tissues available, as shown in this work and in the literature^17,30,39^ on developmental stages and in a lesser, but not negligible, extent on the experimental conditions, or age of the organisms studied. Another limiting factor in such endeavour is the cellular heterogeneity of the tissues. Indeed, tissues are composed of different cell types, and most of them likely contain blood cells that irrigate them. Single-cell RNA sequencing (scRNA-seq) could to be a method of choice to discriminate between cell types and therefore minimize heterogeneity bias. However, scRNA-seq is currently limited in the number of detected transcripts, estimated to only 10–20% of the mRNA molecules actually present^83^, and seems to perform poorly in the detection of low expressed genes, which is the case of LNC. These technical limitations will therefore have to be alleviated for scRNA-seq to be used in LNC detection and modelling.

As a conclusion, this study aims at providing an improved gene annotation enriched in LNC for the chicken species, information on their genomic configuration with respect to PCG and miRNA, and transcription patterns across tissues for the both PCG and LNC of the annotation. Such a catalogue and information associated will be useful to the community to work on LNC, for example, to unveil the molecular chain of causality that links variants located outside coding regions and phenotypes of interest. As an example, Plassais et al*.*^84^ recently demonstrated the causative role of a point mutation in the exon of a LNC, located in divergent configuration with a PCG involved in neural development, in a form of neuropathy. Both genes appeared to be co-expressed, and the point mutation seemed to affect the binding of regulatory elements, leading to the reduction of their expression.

## Methods

### Biological samples used

For the gene modelling (i.e. the computational prediction of transcript structures in the genome), we used 364 RNA-seq samples from three chicken tissues (blood, liver and adipose tissue) with different physiological stages of broilers and layers, for which the raw RNA-seq data are available under the SRP079637 and PRJEB28745, PRJEB34310 and PRJEB34341.

For the expression study, we used 5 tissues (blood, adipose tissue, liver, hypothalamus and embryos) from a Rhode Island Red line with a minimum of 24 biological replicates per tissue (data available under the PRJEB28745). Such a dataset with an average of 80 M reads per biological replicate is referred to as the “5 T dataset” in the Results section. We also used 168 RNA-seq samples from the PRJEB12891, used by Ensembl for annotating the chicken *Gallus_gallus_5* reference genome, version v87 (December 2016) to v94 (October 2018). This dataset was composed of 21 tissues with 8 replicates per tissue with, on average, 15 M of reads per replicate representing one age (16–17 weeks old), one sex (female) of the same J-line strain. It is referred to as the “21 T dataset” in the “Results” section.

### Ethics

All the animal experiments used in the present study were approved by the local ethical committee in animal experimentation of Val de Loire, France (authorization to experiment on living animals n°7740, 30/03/2012) and by the French Ministries of Higher Education and Scientific Research, and of Agriculture and Fisheries (Approval number: 2873–2,015,112,512,076,871). Animal experiments were conducted at the experimental farm PEAT under license number C37-175–1 for animal experimentation, in compliance with the European Union Legislation.

### RNA collection and sequencing

RNA extraction and RNA sequencing were performed as described in Muret et al*.*^22^ and Jehl et al*.*^85^. Briefly, tissues were sampled immediately before (blood) or after slaughter (adipose tissue, liver) and stored appropriately. RNAs were extracted following the kits or reagents manufacturer’s instructions and stored at − 80 °C. Total RNA was quantified using a NanoDrop ND-1000 spectrophotometer (Thermo Scientific, Illkirch, France). The A260/280 and A260/230 ratios were greater than 1.7 in all samples ensuring the RNA purity. The RNA quality was controlled using an Agilent 2100 bioanalyzer (Agilent Technologies France, Massy, France). The RNA integrity numbers were ≥ 7 for the adipose and the whole blood tissue, ≥ 8 for the hypothalamus, ≥ 8.5 for the liver and embryos. The sequencing was conducted in a stranded, paired-end 2 × 150 bp reads, on a HiSeq2000 or HiSeq3000 (Illumina).

### INRAGALG gene modelling

RNA-seq reads were trimmed using cutadapt version 1.8.3. Reads were then mapped on the Ensembl *Gallus_gallus_5* reference genome using STAR^86^ v.2.5.2b, following the multi-sample 2-pass mapping procedure^87^, with the Ensembl v92 GTF file as input file for the generation of genome indices as described in Muret et al*.*^22^. The new transcript models were constructed as described in Foissac et al.^21^. Briefly, after read mapping and CIGAR-based softclip removal, each sample alignment file (BAM file) was processed with Cufflinks 2.2.1 with the max-intron-length (25,000) and overlap-radius (5) options, guided by the reference gene v92 annotation (–GTF-guide option). All cufflinks models were then merged into a single gene annotation using Cuffmerge 2.2. with the –ref-gtf option. Using the 364 RNA-seq samples, we modelled 25,085 putative new genes in addition to the Ensembl genes. To ensure reliability of the models, loci were selected based on their expression and their reproducibility across samples. First, 21,520 genes were selected with an expression greater than or equal to 0.1 TPM (Transcripts Per Million), a common threshold when working on lncRNA genes^88,89^, known to be lowly expressed. However, we observed that some models exceeding this threshold were supported by one or two reads at most, whichever the replicate. Hence, in order to discard such models, we applied a more stringent criterion, consisting in keeping only the models supported by a least five reads in the samples of a given tissue, similarly to de Goede et al*.*^89^. These selection steps resulted in a dataset of 14,760 models, hereafter called *INRAGALG*.

### LNC prediction

The discrimination between coding and long non-coding genes was realized using the FEELnc codpot module of the FEELnc (FlExible Extraction of Long noncoding RNAs, v0.1.0) tool^41,90^, as described in Muret et al*.*^22^. Briefly, FEELnc codpot module calculates a coding potential score (CPS) for the assembled transcripts based on several predictors (such as multi *k*-mer frequencies and Open Reading Frame coverage) incorporated into a random forest algorithm^91^. In order to increase the robustness of the final set of novel lncRNAs and mRNAs, the option –spethres = 0.98 was set. The FEELnc model was trained with the chicken PCG and LNC transcripts from the chicken Ensembl v92 annotation.

### Background noise evaluation

The background noise corresponds to the expression of a set of artificial loci randomly distributed across chicken chromosomes 1 to 33 using the bedtools shuffle function from the BEDTools suite^92^. These artificial loci had the same length distribution as the LNC genes and were positioned at least 5 kb out of the closest known transcribed regions.

### External source pre-treatment and aggregation

*Ensembl* source: the *Gallus_gallus_5* Ensembl v92 reference annotation was downloaded from the Ensembl FTP website^106^ as a GTF file. *INRA* source: the INRA GTF file was obtained and filtered as described in the two previous sections. *ALDB* source: the ALDB^28^ v1.0 database containing 5,752 LNC genes was downloaded from the FANTOM project Zenbu viewer^93^ page, as a BED file in *Gallus_gallus_5* version*. NONCODE* source: the NONCODE v5.0 database containing 9,322 LNC genes was downloaded from the appropriate website^94^, the form of a BED file being in *Galgal4* version. The file was therefore translated into *Gallus_gallus_5* version using the LiftOver tool from UCSC^95^, and the chromosome names were converted into Ensembl chromosome names. *NCBI* source: the NCBI database v103 containing 5,738 LNC genes was downloaded from NCBI FTP website^96^ in the form of a GFF3 file in *Gallus_gallus_5* version. Chromosome names were converted into Ensembl chromosome names. Finally, the GFF3 file was converted into a GTF file as well as the BED files from ALDB and NONCODE sources. *FR-AgENCODE* source: the FR-AgENCODE annotation containing 6,089 LNC genes was obtained from Foissac et al.^21^.

*Aggregation of the sources*: we sequentially added the gene models from each database to a growing catalogue, keeping only the gene models that had no overlap with a gene already present in the growing catalogue. Two gene models were considered as overlapping if one or more of their transcripts were on the same-strand and had one or more nucleotides in common. The gene model overlap between sources was assessed using the bedtools intersect function from the BEDTools suite^92^. The different sources were aggregated to the Ensembl annotation in the order presented in Results, which was determined by calculating the percentage of LNC transcript models having their 5′ extremity within a CAGE peak^38^, suggesting that these transcripts were properly modelled, at least in their 5′-end. Note here that this sequential strategy was chosen since we observed that total aggregation of the overlapping models using the cuffmerge tool tended to create chimeric models composed of one or more known PCG Ensembl genes by dubious LNC models from another database.

### LNC and miRNA classification using FEELnc

Long non-coding transcripts were classified relatively to the closest protein-coding transcript using FEELnc classifier tool^41,90^ with default settings (maximal window of 100 kb). Briefly, using (*i*) the LNC transcript models from the extended annotation and (*ii*) the PCG transcript models, the tool uses a 100 kb sliding window and classifies each LNC transcript using its location and orientation relative to the closest PCG transcript. The results distinguish LNC types (whether genic or intergenic), then subtypes (overlapping, containing and nested subtypes for the genic type, and divergent, convergent and same strand subtypes for the intergenic type) and finally locations (exonic, intronic or upstream, downstream). If no PCG were found within the sliding window, the LNC is considered as “unclassified”. From this transcript level classification, we generated a gene level classification. Generally, the different transcripts of a given LNC gene have the same classification relative to PCG. However, for the rare cases (4%) in which transcripts of a given LNC gene have different classifications relative to one or more PCG, we indicated these conflicts. The FEELnc classifier module was also used to classify the miRNAs present in the Ensembl annotation with respect to their closest LNC for identifying LNC hosting one or several miRNAs.

### Gene expression quantification

FASTQ files were mapped on the Ensembl *Gallus_gallus_5* reference genome using STAR^86^ v.2.5.2b, following the multi-sample 2-pass mapping procedure, with the extended GTF file as input file for the generation of genome indexes step as described in Muret et al*.*^22^. Samples were analysed by tissue. FASTQ files were previously trimmed for Illumina adapter using TrimGalore version 0.4.5. Expression was quantified with RSEM^97^ v.1.3.0, using the extended GTF file at the gene-level^21,22,39^. This workflow is part of a snakemake^98^ pipeline, available at Ref.^99^. Each gene was considered as expressed in at least one tissue of 5 T or 21 T dataset using the criteria TPM ≥ 0.1.

For the 21 T dataset, the 21 tissues and their four letters abbreviations are: bursa of Fabricius (burs), cecal tonsils (cctl), cerebellum (crbl), duodenum (duod), adipose tissue around the gizzard (fatG), harderian gland (hard), heart (hert), ileum (ileu), kidney (kdny), liver (livr), lung (lung), breast muscle (mscB), optical lobe (optc), ovary (ovry), pancreas (pcrs), proventriculus (pvtc), skin (skin), spleen (spln), thymus (thym), thyroid gland (thyr) and trachea (trch). For the 5 T dataset, the 5 tissues, blood, adipose tissue, liver, hypothalamus and embryos, were abbreviated as blod, adip, livr, hypt and embr respectively.

### GTEx data analysis

The version 7 RNA-seq TPM data was downloaded from the GTEx website (https://gtexportal.org/home/). Each gene expression was normalized as log_10_(TPM + 1), and the mean for each of the 53 tissue was calculated. The list of the 53 tissues is available in Supplementary Table S22.

### Tissue-specificity analysis

Tissue-specificity was assessed using the tau (τ) metric^44^, which ranges from 0 (gene expressed at the same level in all tissues) to 1 (gene expressed in exactly one tissue), using the following formula: let $x_{g,t}$ be the expression of the gene $g$, in the tissue $t$, among $T$ tissues. The τ value associated to a gene $g$ is calculated using the following equation:

$$\tau_{g} = \frac{{\mathop \sum \nolimits_{t = 1}^{T} \left( {1 - \hat{x}_{g,t} } \right)}}{T - 1},\quad {\text{where}}\quad \hat{x}_{g,t} = \frac{{x_{g,t} }}{{\mathop {max}\limits_{1 \le t \le T} \left( {x_{g,t} } \right)}}$$

with $x_{g,t}$ being the expression of the gene $g$, in the tissue $t$, among $T$ tissues.

A gene was considered as tissue-specific for τ ≥ 0.95, as done in previous studies^39^, and corresponding to a ratio of 4 between the first and the second tissues with the highest expressions.

### Co-expression analysis

For each LNC:PCG pairs detected, we computed the Spearman correlation (*ρ*) between the expression values across tissues. *P*-values were corrected for multiple testing using the Benjamini–Hochberg method^100^. The false discovery rate was set to 0.05, corresponding to an absolute *ρ* value of 0.55 using the 21 tissues of the PRJEB12891 dataset, referred to as the “21 T dataset” in the Results section.

### GO-terms and KEGG terms analysis

The enrichment analysis of Kyoto Encyclopedia of Genes and Genomes (KEGG) and Gene Ontology (GO) terms in each set of genes of interest was performed using the STRING tool^101^ (https://string-db.org). Only the 1-to-1 human orthologous genes with a standardized HGNC name were submitted for the analysis. To investigate biological functions, we favoured KEGG terms when available, GO terms related to “Biological Process” otherwise. If no such enriched terms were found, we focused on the molecular functions of the genes using the GO “Molecular Function” terms.

### LNC hosting miRNA analysis

For each expressed LNC containing one or more miRNA gene, we calculated the expression correlation between the LNC and all the expressed PCG. The correlation threshold was set at | ρ |≥ 0.8, corresponding to a false discovery rate inferior or equal to 0.05 with the 21 tissues of the PRJEB12891 dataset, referred to as the “21 T dataset” in the Results section. MiRNA expression data were obtained from the Chickspress website^43^ in which 55 tissues and conditions are available, the list of which is provided in Supplementary Table S22. We further selected the LNC for which at least 75% of the correlated PCG had a HGNC and for which the hosted miRNA gene(s) were cited in the literature following a PubMed request of their name. Targets of the miRNAs were screened for in miRTarBase^102^ and mirDB^103^. For the latter, we only considered targets with a target prediction scores ≥ 80, which indicates a high confidence of the prediction.

### Biological validation by RT-PCR and sequencing

The existence of different LNC models was assessed by RT-PCR and sequencing using RNA extracted from different chicken tissues. Each LNC RNA was validated using the tissue in which it was the most expressed. The PCR primers and hybridization temperature used are indicated in Supplementary Table S23 for each analysed LNC. Reverse transcription (RT) was carried out using the high-capacity cDNA archive kit (ThermoFisher Scientific, catalog number: 4368814) according to the manufacturer's protocol. Briefly, reaction mixture containing 2μL of 10 × RT buffer, 0.8μL of 25 × dNTPs, 2μL of 10 × random primers, 1μL of MultiScribe Reverse Transcriptase (50 U/μL), and total RNA (2 μg) was incubated for 10 min at 25 °C followed by 2 h at 37 °C and 5 min at 85 °C. Dilution RT reaction was further used for PCR. 5 µl of cDNA samples were mixed with 5 μl of GoTaq Flexi Buffer 5 × , 2μL of MgCl_2_ solution (25 mM), 0.125 μl of GoTaq DNA Polymerase (5u/µl) (Promega, catalog number: M891), 0.5 μL of dNTPs 10 mM, 12.5 µl H20 and 1.25 µl of specific reverse and forward primers at 10 µM. Reaction mixtures were then incubated in a T100 Thermal cycler (Bio-Rad, Marne la Coquette, France). The amplification products are then deposited on a 2% agarose gel and sent for sequencing (Genoscreen) to verify their location in the chicken genome. LNC were considered as validated if a PCR amplification was observed and their location in the genome was the expected one.

### Update of the catalogue in GRCg6a reference genome using the associated Ensembl v100 annotation

Transcripts sequences were extracted from the Gallus_gallus-5.0 FASTA file using gffread^104^ v0.11.0 and mapped to the GRCg6a assembly sequence using GMAP^105^ 2015-11-20 with default parameters. If none of a gene's transcripts had a unique position (mapped on different chromosomes or on different strands), the gene and its transcripts were removed from the annotation. The genes from our catalogue in Gallus_gallus-5.0 coordinates for which even one transcript overlapped an Ensembl gene from the v100 annotation were removed. This insures that the annotation that we provide in GRCg6a positions is composed of the full Ensembl v100 annotation, enriched in LNC gene models from the present catalogue. This GTF generated in coordinates GRCg6a, as well as the GTF in Gallus_gallus-5.0 coordinates can be found the FR-AgENCODE website (http://www.fragencode.org/overview.html).

## Supplementary information

Supplementary Information.

Supplementary Information 2.

Supplementary Information 3.

Supplementary Information 4.

Supplementary Information 5.

## Data Availability

The raw transcriptomic data used in this work are available on ENA under accession numbers: PRJEB28745, PRJEB34310 and PRJEB34341, and on SRA under Accession No. SRP079637.
