# Author Correction: An integrative atlas of chicken long non-coding genes and their annotations across 25 tissues

**DOI:** 10.1038/s41598-021-89158-8

**Published:** 2021-04-28

**Authors:** Frédéric Jehl, Kévin Muret, Maria Bernard, Morgane Boutin, Laetitia Lagoutte, Colette Désert, Patrice Dehais, Diane Esquerré, Hervé Acloque, Elisabetta Giuffra, Sarah Djebali, Sylvain Foissac, Thomas Derrien, Frédérique Pitel, Tatiana Zerjal, Christophe Klopp, Sandrine Lagarrigue

**Affiliations:** 1 PEGASE UMR 1348, INRA, AGROCAMPUS OUEST, 35590 Saint-Gilles, France; 2 SIGENAE Platform, INRA, 31326 Castanet-Tolosan, France; 3 Genotoul, INRA, US 1426 GeT PlaGe, Castanet Tolosan, France; 4 GenPhySE UMR 1388, INRA, INPT, ENVT, Université de Toulouse, 31326 Castanet-Tolosan, France; 5 GABI UMR 1313, INRA, AgroParisTech, Université Paris-Saclay, 78350 Jouy-en-Josas, France; 6 IRSD, Université de Toulouse, INSERM, INRA, ENVT, UPS, Toulouse, France; 7 IGDR UMR 6290, CNRS, Univ Rennes, 35000 Rennes, France

Correction to: *Scientific Reports* 10.1038/s41598-020-77586-x, published online 24 November 2020

The original version of this Article contained an error in Affiliation 3, which was incorrectly given as ‘GENOTOUL Platform, INRA, 31,326, Castanet-Tolosan, France’. The correct affiliation is listed below:

Genotoul, INRA, US 1426 GeT PlaGe, Castanet Tolosan, France.

The original Article and accompanying Supplementary Information files have been corrected.

