# Supplementary material for: An integrative atlas of chicken long non-coding genes and their annotations across 25 tissues
Source: Sci Rep. 2020 Nov 24;10:20457. doi: 10.1038/s41598-020-77586-x (PMC7686352; doi:10.1038/s41598-020-77586-x)
Supplement: Supplementary file 3 — Supplementary Information 3. [file 41598_2020_77586_MOESM3_ESM.docx]

**An integrative atlas of chicken long non-coding genes and their annotations across 25 tissues**

Frédéric Jehl^1,$^, Kévin Muret^1,$^, Maria Bernard^2,$^, Morgane Boutin^1^, Laetitia Lagoutte^1^, Colette Désert^1^, Patrice Dehais^2^, Diane Esquerré^3^, Hervé Acloque^4^, Elisabetta Giuffra^5^, Sarah Djebali^6^, Sylvain Foissac^4^, Thomas Derrien^7^, Frédérique Pitel^4^, Tatiana Zerjal^5^, Christophe Klopp^2,*^ and Sandrine Lagarrigue^1,*^

^1^PEGASE UMR 1348, INRA, AGROCAMPUS OUEST, 35590 Saint-Gilles, France

^2^SIGENAE Platform, INRA, 31326 Castanet-Tolosan, France

^3^Genotoul, INRA, US 1426 GeT PlaGe, Castanet Tolosan, France

^4^GenPhySE UMR 1388, INRA, INPT, ENVT, Université de Toulouse, 31326 Castanet-Tolosan, France

^5^GABI UMR 1313, INRA, AgroParisTech, Université Paris-Saclay, 78350 Jouy-en-Josas, France

^6^IRSD, Université de Toulouse, INSERM, INRA, ENVT, UPS, Toulouse, France.

^7^IGDR UMR 6290, Univ Rennes, CNRS, 35000, Rennes, France

^*^Corresponding author

**GENERAL INFORMATIONS ON THE FEATURE**

**chr**: chromosome

**start**: genomic coordinate of the start of the feature (gene/transcript)

**end**: genomic coordinate of the end of the feature

**strand**: strand of the feature (+ or -)

**gnId**: unique identifier of the gene corresponding to the feature

**tpId**: unique identifier of the transcript for the transcripts level annotation,

semi-column-separated unique identifiers of all the transcripts corresponding to the gene for the gene level annotation

**source**: source of the feature


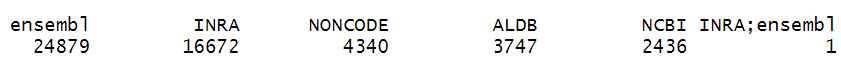


**Figure 1**. Number of models from each source. The “INRA;ensembl” source arises from SCD gene model which was corrected to include a nearby LNC that was shown to be the same gene (Muret *et al.* 2019,BMC genomics).

**version**: version of the database used

**gnBiotype**: biotype of the gene in a standardized way

lnc = long non-coding genes

mir = miRNA

mis = misc_RNA. For INRA source, the “mis” genes correspond to the Transcript of Unknown Coding Potential (TUCP) from FEELnc’ “codpot” module.

mtr = Mt_rRNA

mtt = Mt_tRNA

pcg = protein coding gene

pse = pseudogene

rbz = ribozyme

rrn = rRNA

sca = scaRNA

sno = snoRNA

snr = snRNA

srn = sRNA


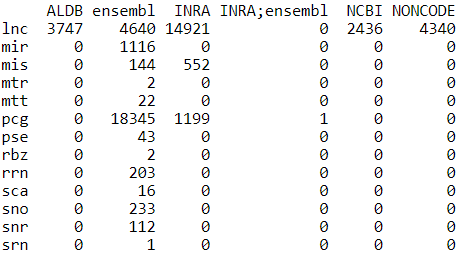


**Figure 2**. Number of each genes biotypes from each source.

**tpBiotype**: biotype of the transcript corresponding to the feature for the transcripts level annotation, semi-column-separated biotype of all the transcripts corresponding to the gene for the gene level annotation

**gnName**: name of the gene corresponding to the feature, after the following decision rule:

= hgncHsa1to1 > Chicken HGNC (hgncGga) > Mouse 1to1 HGNC (hgncMmu1to1) > Human 1to1 wikigene (wikigeneHsa1to1) > wikigeneGga > wikigeneMmu1to1 > Gene name as found in the field “gene_name” of the Ensembl GalGal5 GTF, v94 (gnNameGga) > Human 1tomany HGNC (hgncHsaXtoMany) > Mouse 1tomany HGNC (hgncMmuXtoMany) > Human 1tomany wikigene (wikigeneHsaXtoMany) > Mouse 1tomany wikigene (wikigeneMmuXtoMany) > gene ID (as in **gnId** column).

Note: all the 1-to-many and many-to-Many names are followed by “_XtoMany”

**Table 1:** number of genes with HGNC identifier, LOC, 1-to-Many or Many-to-Many and gene ID in the **gnName** column.

| **Type of name** | **Number of genes** |
| --- | --- |
| HGNC identifier | 15747 |
| LOCxxx name | 911 |
| Containing “_XtoMany “ | 1025 |
| Gene ID (ENSGALG, INRAGALG, etc.) | 31956 |

**gnNameDesc**: full name of the gene associated to the feature, originating from the same database as the **gnName** column.

**gnNameGga**: name associated to the gene in Ensembl Galgal5 (from BioMart)

**hgncGga**: HGNC symbol associated to the gene (from BioMart)

**wikigeneGga**: wikigene symbol associated to the gene (from BioMart)

**hgncHsa1to1**: HGNC symbol of the 1-to-1 human’s orthologue of the gene (from BioMart)

**wikigeneHsa1to1**: wikigene symbol of the 1-to-1 human’s orthologue of the gene (from BioMart)

**hgncMmu1to1**: HGNC symbol of the 1-to-1 mouse’s orthologue of the gene (from BioMart)

**wikigeneMmu1to1**: wikigene symbol of the 1-to-1 mouse’s orthologue of the gene (from BioMart)

**gnNameDescGga**: full name associated to the gene in Ensembl Galgal5 (from BioMart)

**gnNameDescHsa1to1**: full name of the 1-to-1 human’s orthologue of the gene (from BioMart)

**gnNameDescMmu1to1**: full name of the 1-to-1 mouse’s orthologue of the gene (from BioMart)

**gnNameDescHsaXtoMany**: full name of the X-to-many human’s orthologue of the gene (from BioMart)

**gnNameDescMmuXtoMany**: full name of the X-to-many mouse’s orthologue of the gene (from BioMart)

**orthHsaType**: type of orthology between the chicken genes and the human genes (from BioMart)


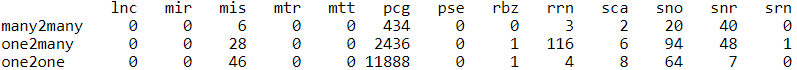


**Figure 3:** Number of genes with a 1-to-1, 1-to-many and many-to-many ortholog in human, as a function of the gene simple biotype (**gnBiotype**)

**orthMmuType**: type of orthology between the chicken genes and the mouse genes (from BioMart)


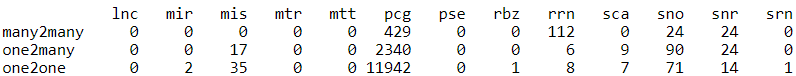


**Figure 4:** Number of genes with a 1-to-1, 1-to-many and many-to-many ortholog in mouse, as a function of the gene simple biotype (**gnBiotype**)

**orthHsaGnId**: Identifier of the human gene ID associated to the chicken gene (from BioMart)

Note: all the 1tomany and manyToMany ID are followed by “_XtoMany”

**orthMmuGnId**: Identifier of the mouse gene ID associated to the chicken gene (from BioMart)

Note: all the 1tomany and manyToMany ID are followed by “_XtoMany”

**hgncHsaXtoMany:** HGNC symbol of the X-to-many human’s orthologue of the gene (from BioMart)

**hgncMmuXtoMany:** HGNC symbol of the X-to-many mouses’s orthologue of the gene (from BioMart)

**wikigeneHsaXtoMany:** Wikigene symbol of the X-to-many human’s orthologue of the gene (from BioMart)

**wikigeneMmuXtoMany:** Wikigene symbol of the X-to-many mouse’s orthologue of the gene (from BioMart)

**exNb**: number of exon(s) of the of the transcript(s) associated to the gene, in the format “**minimum** number of exon” (= number of exons of the transcript with the least exons) ; “**median** of the number of exon of the transcript(s)” ; “**maximum** number of exons” (= number of exons of the transcript with the most exons), semi-column-separated

**exSz:** Size of the exon(s) of the of the transcript(s) associated to the gene, in the format “**minimum** length of exon” (= smallest exon) ; “**median** size of exon of the transcript(s)” ; “**maximum** size of exon” (= longest exon), semi-column-separated

**inSz:** Size of the intron(s) of the of the transcript(s) associated to the gene, in the format “**minimum** length of intron” (= smallest intron); “**median** size of intron of the transcript(s)” ; “**maximum** size of intron” (= longest intron), semi-column-separated

**tpSz:** Size of the transcript(s) associated to the gene. Calculated as the sum of the length of all the exons associated to each transcript. Format: “**minimum** length of transcript” (= smallest transcript) ; “**median** size of transcript(s)” ; “**maximum** size of transcript” (= longest transcript), semi-column-separated

**nbTp**: number of transcript(s) associated to the gene.

**gnSz**: size of the gene. Calculated as the median of the size of all the transcripts associated to the gene.

**LONG NON-CODING GENES ANNOTATION WITH RESPECT TO THE CLOSEST PROTEIN-CODING GENE**

The column following concerns the FEELnc class annotation of the LNC (feelLnc) with respect to the nearest protein coding gene (feelLncPcg).

- feelLncPcgClassName, feelLncPcgClassType, feelLncPcgGnId, feelLncPcgGnName, feelLncPcgGnDist,

**feelLncPcgClassName:** Abbreviation of the FEELnc classification of the LNC with respect to the closest PCG

To transfer the FEELnc information from the transcript level to the gene level, an order of importance has to be decided.

The class names are composed of three parts:

- the first part (8 letters) is composed of the main class type. For the genic classes: lncgSSex, lncgSSin, lncgASex, lncgASin. For the intergenic classes: lincDivg, lincSSup, lincSSdw, lincConv.
- the second part (4 letters) concerns only the genic classes without subtype conflicts (see below), we add one of the three subtypes: Nested (Nest), Overlapping (Ovlp) or Containing (Cont)
- the third part (_n.n.n or _n.1.n) indicates that there are conflicts between annotation due to several PCGs related to the LNC locus.

Conflicts cases are of two types: the cases in which there are more than 1 annotation relative to one unique PCG (as indicated by the “n” in the middle and “1” at the end of “n.n.1”), and the case in which there more than 1 or more annotation relative to more than 1 PCG (“n.X.n” in the **feelLncPcgClassType** column). In these cases, we prioritized the annotation in the column « feelLncPcgClassName», which gives only 1 class per gene.

→ n.n.1 case: Genics have priority over intergenics (lncg > linc).

Among the genic, exonics have priority over intronics.

Among exonics and intronics, the subtypes nested / containing / overlapping have the same importance. They are kept if they do not produce conflicts and are removed if there are 2 or more subtypes.

→ n.X.n case: Same order of priority as previously (n.n.1 case).

Concerning the intergenics, there can be annotation conflicts between the several PCGs: the LNC can be classified as lincDivg with one PCG and lincConv with another one PCG (see figure 1 for an example). We prioritize the classes as following: Divg > SS > Conv. Same-strand have priority over Conv because it could suggest an error in the modelization: the LNC could be a 5’-part or 3’-part of the PCG. Between the same strand up and down (lincSSup and lincSSdw), we choose the closest. The third part of the class name of these genes is either “_n.n.n” or “_n.1.n”.


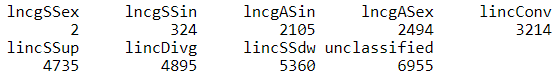


**Figure 5:** Simplified class name present in the file for the LNC:PCG pairs

**feelLncPcgClassType:** gives information on three fields separated by a dot “.” (X_1_.X_2_.X_3_) about the classification done by FEELnc of the LNC transcript relatively to the closest PCG transcript (= LNC:PCG pair):

- X_1_: number of transcripts of the LNC gene: “1” if 1 transcript, “n” if more than one transcript,
- X_2_: number of feelnc class(es) associated to the LNC:PCG pair: “1” if 1 class, “n” if more than one class (the “unclassified” class does *not* count),
- X_3_: number of PCG gene(s) concerned by this (these) annotation(s): “1” if 1 PCG gene, “n” if more than one PCG gene.

1.1.1: the LNC has 1 transcript, with 1 annotation associated to 1 PCG.

n.1.1: the LNC has several transcripts, all with the same annotation associated to the same PCG.

n.n.1: the LNC has several transcripts, with different annotations associated to the same PCG.

n.n.n: the LNC has several transcripts, with different annotations associated to different PCG.

n.1.n: the LNC has several transcripts, all with the same annotation but associated to different PCG.

unclassified: the LNC is either alone in a contig (beginning by AADN. or KQ), or no interactions were found within the 100 000 pb sliding window used by FEELnc.


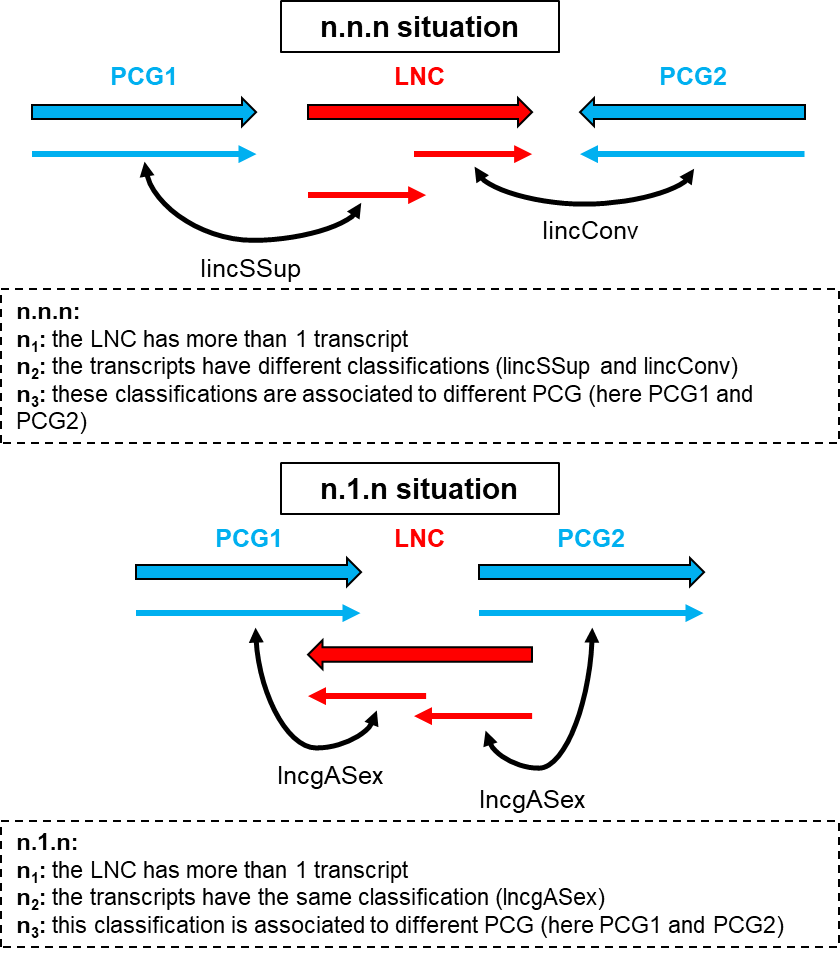


**Figure 3**. examples of configurations corresponding to a “n.n.n” type (top) or a “n.1.n” type (bottom)


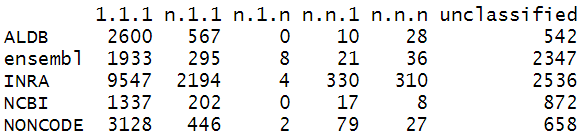


**Figure 4**. Repartition of the different classification types across databases for the LNC:PCG classification

**feelLncPcgGnId**: Unique identifier of the protein-coding gene relatively to which a LNC gene is classified by FEELnc

**feelLncPcgGnName**: Name of the protein-coding gene relatively to which a LNC gene is classified by FEELnc

**feelLncPcgGnDist**: Distance (in bp), as calculated by FEELnc, between the protein-coding gene relatively to which a LNC gene is classified by FEELnc and the LNC gene.

**PROTEIN-CODING GENES ANNOTATION WITH RESPECT TO THE CLOSEST PROTEIN-CODING GENE**

**feelPcgPcgClassName**: Abbreviation of the FEELnc classification of the PCG with respect to the closest PCG. Abbreviations are similar to those of LNC:PCG classification.

**feelPcgPcgClassType**: gives information on three fields separated by a dot “.” (X1.X2.X3) about the classification done by FEELnc of the PCG transcript relatively to the closest PCG transcript, similarly to the LNC:PCG classification.


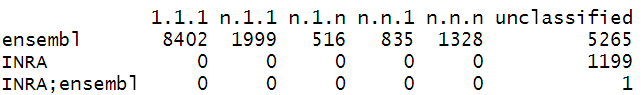


**Figure 5**. Repartition of the different classification types across databases for the PCG:PCG classification. The “INRA;ensembl” source arises from SCD gene model which was corrected to include a nearby LNC that was shown to be the same gene (Muret *et al.* 2019,BMC genomics).

**feelPcgPcgGnId**: unique identifier of the coding gene relatively to which another protein-coding gene is classified by FEELnc.

**feelPcgPcgGnName**: name of the protein-coding gene relatively to which a PCG gene is classified by FEELnc

**feelPcgPcgGnDist**: distance (in bp) between the coding gene relatively to which a protein-coding gene is classified by FEELnc and the PCG

**MICRO RNA AND SMALL RNA GENES ANNOTATION WITH RESPECT TO THE CLOSEST LONG NON-CODING GENE**

**feelMirLncClassName**: Abbreviation of the FEELnc classification of the miRNA with respect to the closest LNC. Abbreviations are similar to those of LNC:PCG classification. From the point of view of procedure, we began by classifying the miRNA with respect to the LNC, obtaining one classification per miRNA (i.e., one LNC associated to each miRNA). We then reversed this classification in order to associate to each LNC every miRNA that was associated to the LNC.

**feelMirLncGnId**: Unique identifier of the miRNA relatively to which a LNC is classified by FEELnc

**feelMirLncGnName**: Name of the miRNA relatively to which a LNC gene is classified by FEELnc.

**feelMirLncGnDist**: Distance (in bp), as calculated by FEELnc, between the miRNA gene relatively to which a LNC gene is classified by FEELnc and the LNC gene

**feelSmlLncClassName**: Abbreviation of the FEELnc classification of the small RNA with respect to the closest LNC. Abbreviations are similar to those of LNC:PCG classification. From the point of view of procedure, we began by classifying the small RNA with respect to the LNC, obtaining one classification per small RNA (i.e., one LNC associated to each small RNA). We then reversed this classification in order to associate to each LNC every small RNA that was associated to the LNC

**feelSmlLncGnId**: Unique identifier of the small RNA relatively to which a LNC is classified by FEELnc

**feelSmlLncGnName**: Name of the small RNA relatively to which a LNC gene is classified by FEELnc.

**feelSmlLncGnDist**: Distance (in bp), as calculated by FEELnc, between the small RNA gene relatively to which a LNC gene is classified by FEELnc and the LNC gene

**DETAILS OF THE BY TRANSCRIPTS ANNOTATION OF THE LNC, PCG, MIRNA AND SMALL RNA WITH RESPECT TO THE CLOSEST RELEVANT GENE**

In the following columns, there are as many fields (Class Name, gene ID, distance, etc.) as transcripts, and the fields are separated by semi-columns “;”.

**feelLncPcgClassNameByTp**: abbreviation of the FEELnc classification for each transcript of the LNC with respect to the closest PCG. Abbreviations are similar to those of LNC:PCG classification at the gene level.

**feelLncPcgGnIdByTp**: unique identifier of the protein-coding gene relatively to which each transcript of the LNC is classified by FEELnc

**feelLncPcgGnNameByTp**: name of the protein-coding gene relatively to which each transcript of the LNC is classified by FEELnc

**feelLncPcgGnDistByTp**: distance (in bp) between each transcript of the LNC and the protein-coding gene relatively to which each transcript of the LNC is classified by FEELnc

**feelPcgPcgClassNameByTp**: abbreviation of the FEELnc classification for each transcript of the PCG with respect to the closest PCG. Abbreviations are similar to those of LNC:PCG classification at the gene level.

**feelPcgPcgGnIdByTp**: name of the protein-coding gene relatively to which each transcript of the PCG is classified by FEELnc

**feelPcgPcgGnNameByTp**: name of the protein-coding gene relatively to which each transcript of the PCG is classified by FEELnc

**feelPcgPcgGnDistByTp**: distance (in bp) between each transcript of the PCG and the protein-coding gene relatively to which each transcript of the LNC is classified by FEELnc

**feelMirLncClassNameByTp**: abbreviation of the FEELnc classification for each transcript of the miRNA with respect to the closest PCG. Abbreviations are similar to those of LNC:PCG classification at the gene level.

**feelMirLncGnIdByTp**: name of the protein-coding gene relatively to which each transcript of the miRNA is classified by FEELnc

**feelMirLncGnNameByTp**: name of the protein-coding gene relatively to which each transcript of the miRNA is classified by FEELnc

**feelMirLncGnDistByTp**: distance (in bp) between each transcript of the miRNA and the protein-coding gene relatively to which each transcript of the LNC is classified by FEELnc

**feelSmlLncClassNameByTp**: abbreviation of the FEELnc classification for each transcript of the small RNA with respect to the closest PCG. Abbreviations are similar to those of LNC:PCG classification at the gene level.

**feelSmlLncGnIdByTp**: name of the protein-coding gene relatively to which each transcript of the small RNA is classified by FEELnc

**feelSmlLncGnNameByTp**: name of the protein-coding gene relatively to which each transcript of the small RNA is classified by FEELnc

**feelSmlLncGnDistByTp**: distance (in bp) between each transcript of the small RNA and the protein-coding gene relatively to which each transcript of the LNC is classified by FEELnc

**IDENTIFIERS OF THE GENES THAT OVERLAPPED THE POSITION OF AN INRAGAL GENE**

**modelOverlappingWithINRAGALG**: When relevant, identifier of the gene from one of the databases (ALDB, NCBI NONCODE) that had at least a 1-pb overlap with a INRAGALG gene during the building of the GTF.

⇒ 2556 gene identifiers.

**IDENTIFIERS OF THE GENES THAT OVERLAPPED THE POSITION OF AN INRAGAL GENE**

**isGeneInGRCg6a**: Indicates whether or not the gene identifier is also present in the extended GTF built from Ensembl v100 annotation of the GRCg6a chicken genome assembly that comprises genes present in our present catalogue that did not overlap genes from the Ensembl v100 annotation.
