# Supplementary material for: An integrative atlas of chicken long non-coding genes and their annotations across 25 tissues
Source: Sci Rep. 2020 Nov 24;10:20457. doi: 10.1038/s41598-020-77586-x (PMC7686352; doi:10.1038/s41598-020-77586-x)
Supplement: Supplementary file 4 — Supplementary Information 4. [file 41598_2020_77586_MOESM4_ESM.docx]

**An integrative atlas of chicken long non-coding genes and their annotations across 25 tissues**

Frédéric Jehl^1,$^, Kévin Muret^1,$^, Maria Bernard^2,$^, Morgane Boutin^1^, Laetitia Lagoutte^1^, Colette Désert^1^, Patrice Dehais^2^, Diane Esquerré^3^, Hervé Acloque^4^, Elisabetta Giuffra^5^, Sarah Djebali^6^, Sylvain Foissac^4^, Thomas Derrien^7^, Frédérique Pitel^4^, Tatiana Zerjal^5^, Christophe Klopp^2,*^ and Sandrine Lagarrigue^1,*^

^1^PEGASE UMR 1348, INRA, AGROCAMPUS OUEST, 35590 Saint-Gilles, France

^2^SIGENAE Platform, INRA, 31326 Castanet-Tolosan, France

^3^Genotoul, INRA, US 1426 GeT PlaGe, Castanet Tolosan, France

^4^GenPhySE UMR 1388, INRA, INPT, ENVT, Université de Toulouse, 31326 Castanet-Tolosan, France

^5^GABI UMR 1313, INRA, AgroParisTech, Université Paris-Saclay, 78350 Jouy-en-Josas, France

^6^IRSD, Université de Toulouse, INSERM, INRA, ENVT, UPS, Toulouse, France.

^7^IGDR UMR 6290, Univ Rennes, CNRS, 35000, Rennes, France

^*^Corresponding author


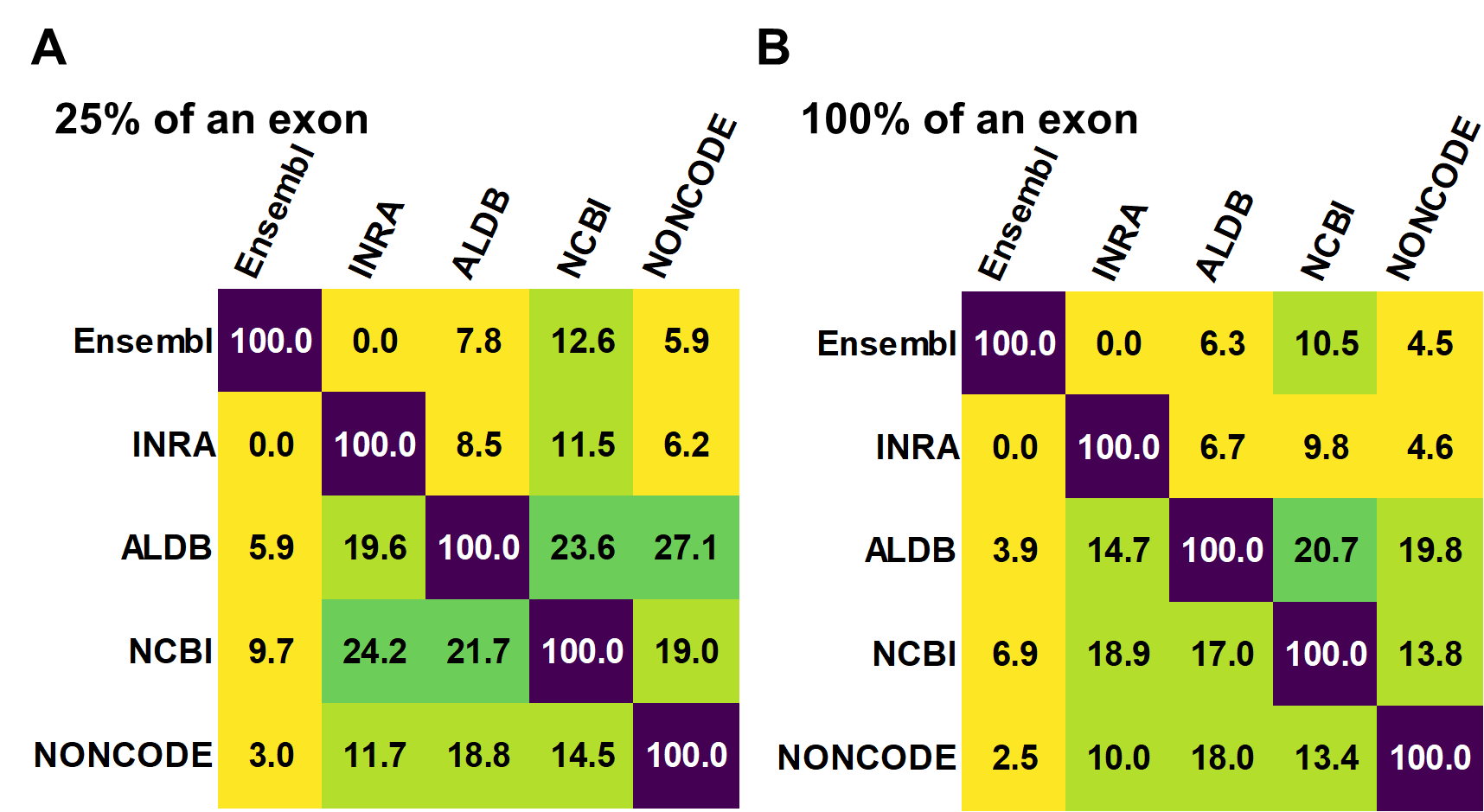


**Supplementary Figure S1**. Heatmap of the overlap between databases expressed in % of LNC (in line) shared among databases (in column), using 25% of an exon **(A)** and 100% of an exon **(B)** overlap


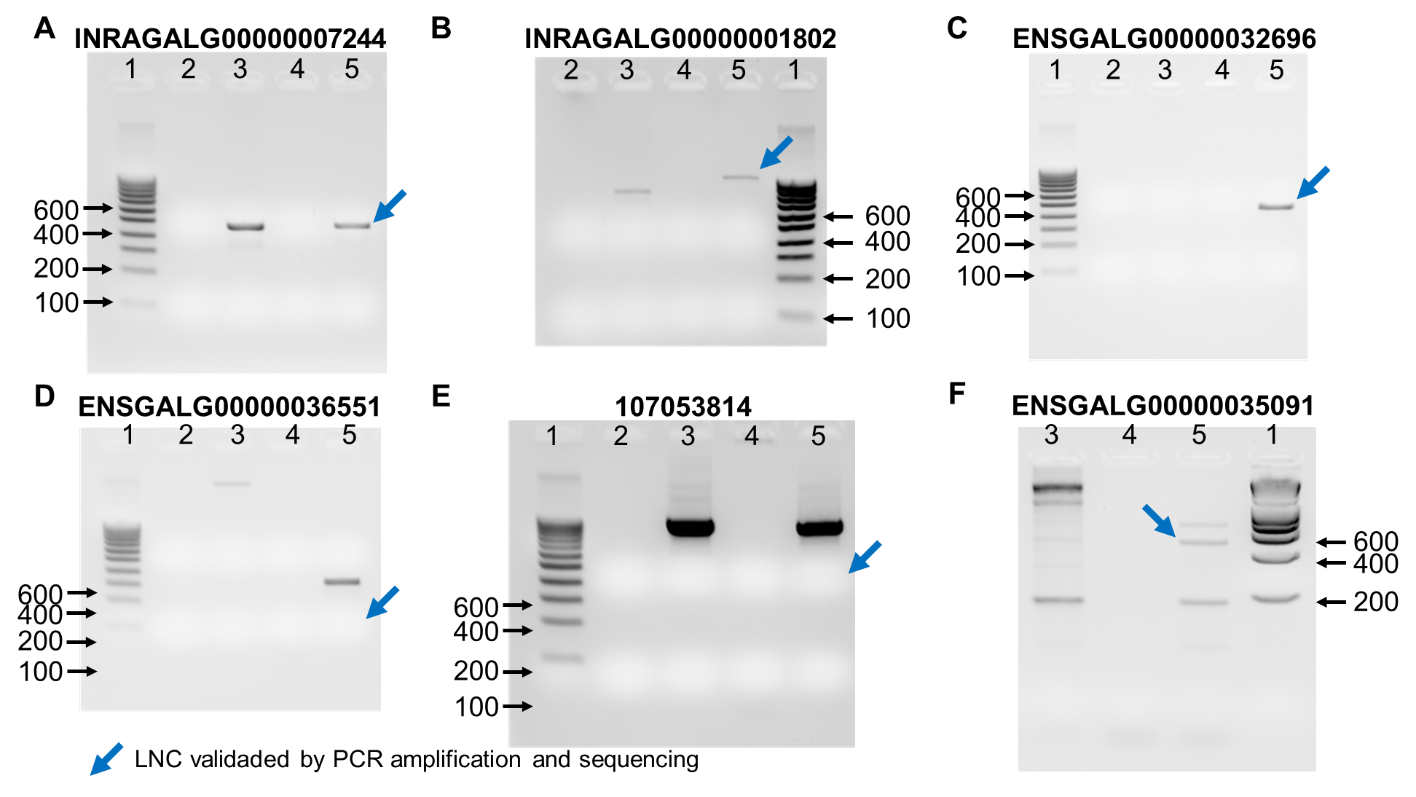


**Supplementary Figure S2**. Experimental validation of 6 LNC presented in main text. **(A)** INRAGALG00000007244, divergent of *PXDC1*. **(B)** INRAGALG00000001802, host of mir-155. **(C)** ENSGALG00000032696, divergent of *LHX5*. **(D)** ENSGALG00000036551, divergent of *TBX4*. **(E)** 107053814, divergent of *HOXD3*. **(F)** ENSGALG00000035091, divergent of *SOX1*.

Number above each gel correspond to: 1: ladder; 2: PCR negative control; 3: PCR using DNA; 4: PCR using RNA; 5: PCR using cDNA. Arrows next to the ladder indicate the size of the ladder’s fragments. Blue arrows next to the band indicate the LNC that was validated by PCR amplification and sequencing.


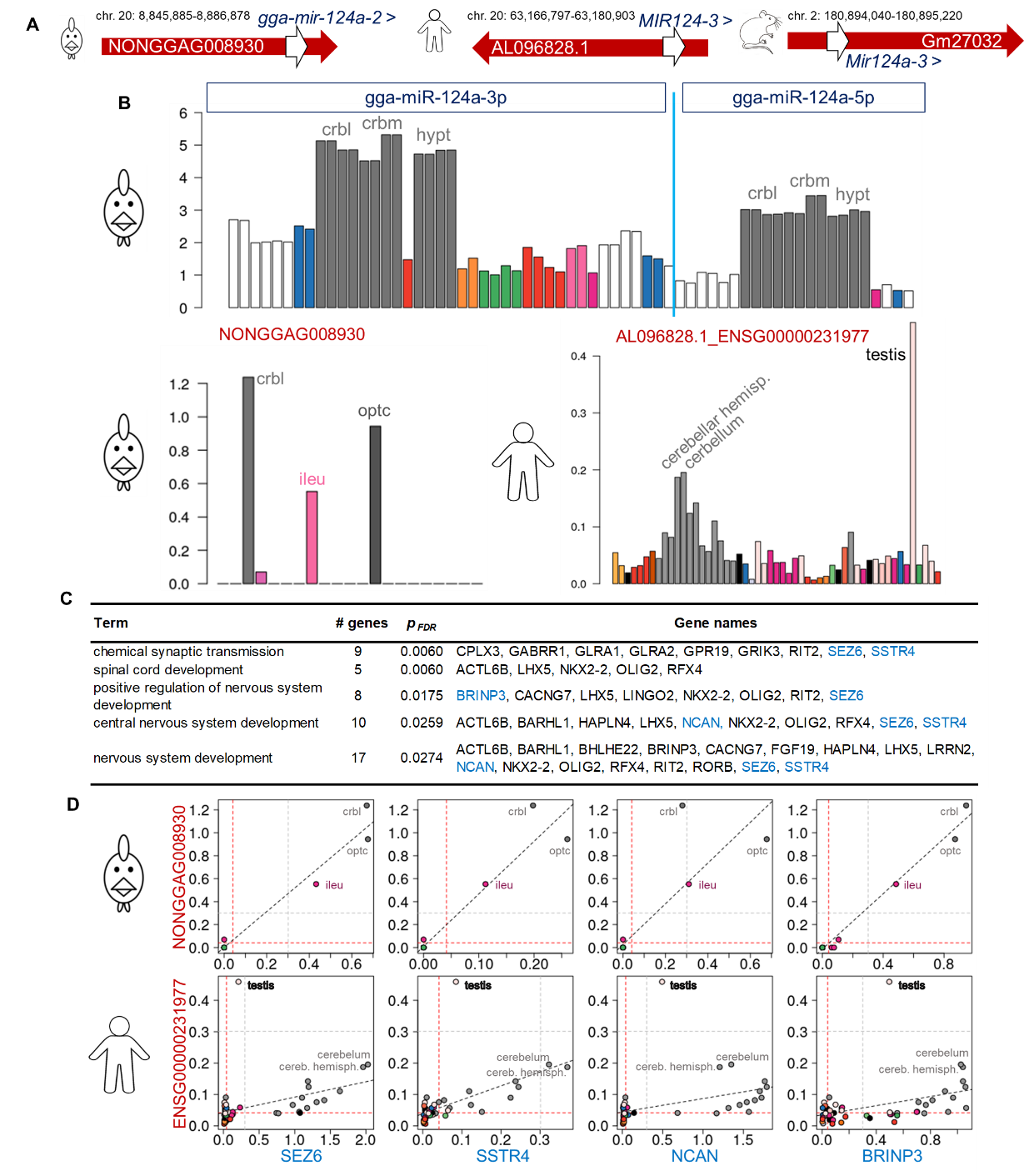


**Supplementary Figure S3. Conservation of genomic location and function of a miR host LNC across species. (A)** NONGGAG008930 hosts gga-mir-124a-2 and might be conserved in human (AL096828.1) and mouse (Gm27032). **(B)** gga-mir-124a-2 (left) and its host LNC, NONGGAG008930 (middle), are mostly expressed in immunity-related tissues in chicken, similarly to AL096828.1 in human (right). Gga-mir-124a-2 expression is expressed in log_10_(FPKM + 1), NONGGAG008930 and AL096828.1 expressions are expressed in log_10_(TPM + 1) **(C)** Top 5 enriched KEGG terms supported by more than 5 genes associated to the PCG correlated to NONGGAG008930. PCG in blue are used in next panel. **(D)** Co-expression of four PCG from previous panel with NONGGAG008930 in chicken (top) or AL096828.1 in human (bottom).

Among the 89 PCG, we found 3 targets of the chicken or human miRNAs:

*hsa-miR124-3p*: *RFX4* detected as a target (target score = 98),

*has-miR124-5p*: no target detected (target score ≥ 80),

*gga-miR-124a-3p*: *RFX4* (target score = 93) was also detected as a target, along with *NOL4* (target score = 92).

*gga-miR-124a-5p*: *NOL4* (target score ≥ 80) was also detected as a target, along with *RIT2* (target score ≥ 80).


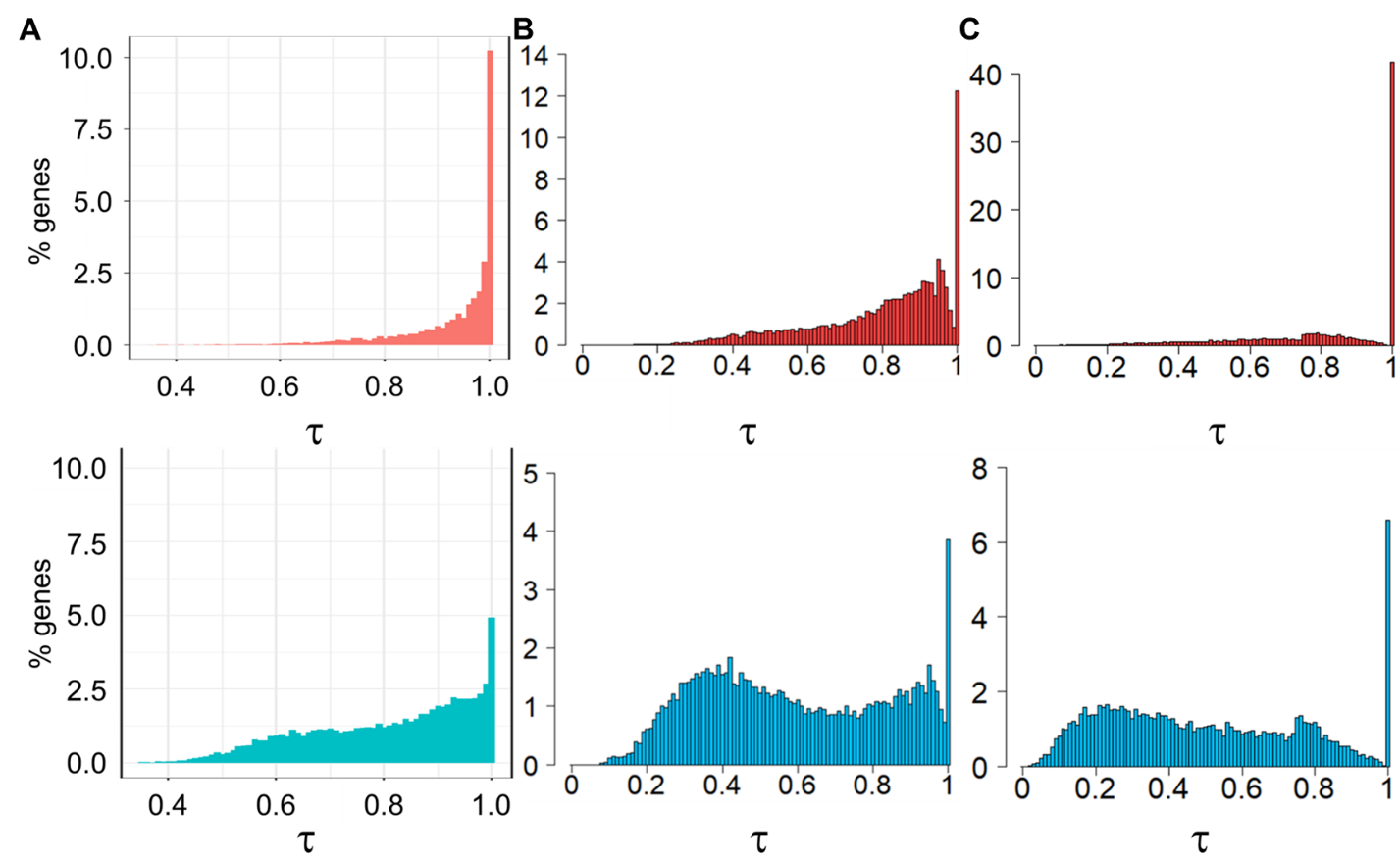


**Supplementary Figure S4. Distribution of the τ values in dog (A), and in chicken 21T (B) and chicken 5T (C) projects.** Top, in red, τ values associated to LNC. Bottom, in blue, τ values associated to PCG

**
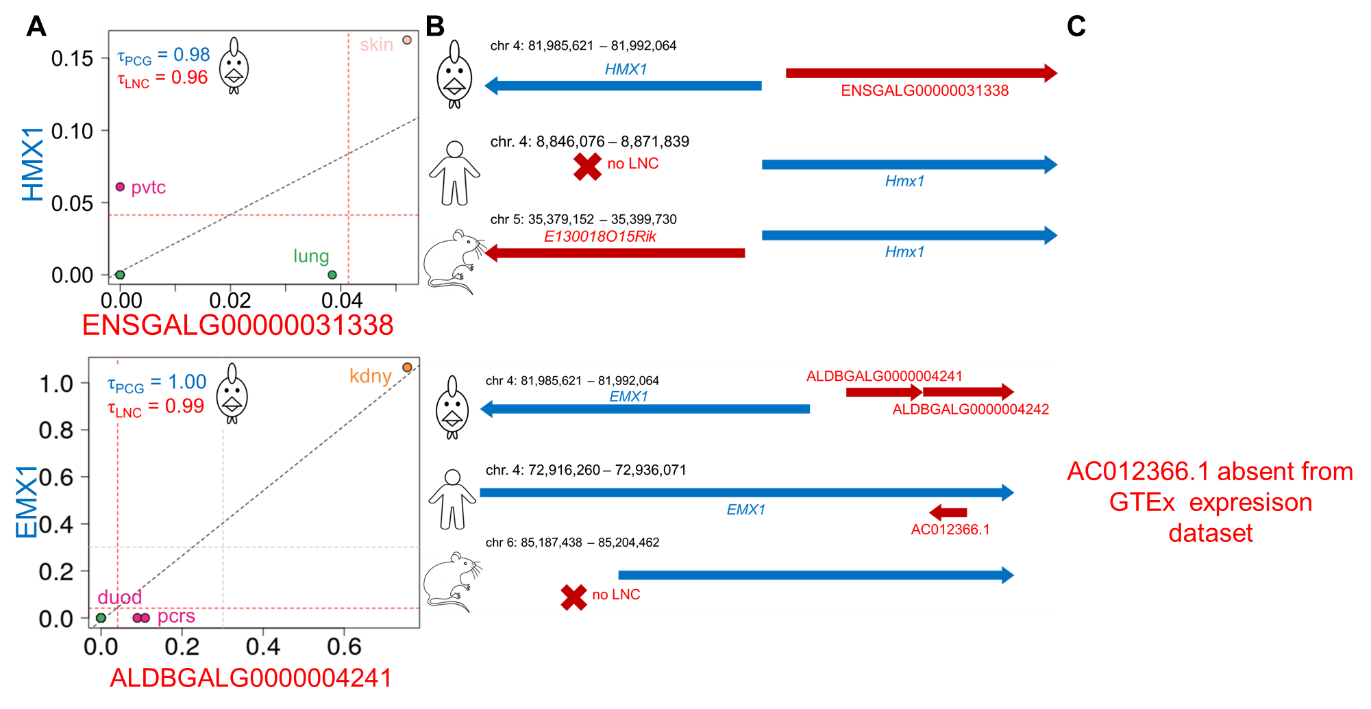
**

**Supplementary Figure S5.** Tissue expression in chicken **(A)** for the 2 LNC:PCG pairs with similar genomic configurations between the two species **(B)** not presented in Main Text Figure 6. **(A)** log10(TPM+1) expression of the LNC (X-axis) and the PCG (Y-axis) (top) for chicken across the 21 tissues of the 21T dataset for the two chicken divergent pairs for which both members are tissue-specific in the same tissue. **(B)** Genomic configuration in three species of the LNC:PCG pairs in chicken, human and mouse. In red the LNC, in blue the PCG. **NB:** Corresponding LNC in human was either not found (for LNC:*HMX1* pair) or absent from the GTEx expression dataset (for LNC:*EMX1* pair).
